# A Synthetic β‐Mannan Epitope Enables Immunization and Detection of *Candida auris*

**DOI:** 10.1002/anie.9466991

**Published:** 2026-08-11

**Authors:** Emelie E. Reuber, Arnab Pradhan, Ian Leaves, Rajat K. Singh, Emer Hickey, Carola Geiler, Neil A. R. Gow, Alistair J. P. Brown, Peter H. Seeberger

**Affiliations:** ^1^ Department of Chemistry and Biochemistry Freie Universität Berlin Berlin Germany; ^2^ Department of Biomolecular Systems Max Planck Institute of Colloids and Interfaces Potsdam Germany; ^3^ MRC Centre for Medical Mycology University of Exeter Exeter UK

**Keywords:** antibodies, *Candida*, carbohydrates, glycoconjugates, vaccines

## Abstract

*Candida auris* is an emerging multidrug‐resistant fungal pathogen for which neither vaccines nor rapid diagnostics are available. Defined fungal glycan epitopes offer a chemically precise route toward both immunization and detection. Here, we show that a synthetic β‐mannan tetrasaccharide, β‐(1,2)Man‐α‐(1,2)Man‐α‐(1,2)Man‐α‐(1,2)Man, functions as a lead epitope against *C. auris*. Conjugation of this glycan to the carrier protein CRM_197_ afforded a glycoconjugate that elicited epitope‐selective immunoglobulin M (IgM) and immunoglobulin G (IgG) responses and reduced fungal burden in kidneys and spleen in a murine disseminated infection model. Monoclonal IgG1 antibodies raised against the same synthetic glycan displayed structure‐dependent recognition of oligomannosides and *Candida* cells and enabled passive immunization, resulting in reduced fungal burden in the spleen. One antibody enabled the development of a prototype lateral flow assay for rapid detection of multiple *Candida* species. The defined synthetic β‐mannan epitope connects glycoconjugate immunization with antibody‐based recognition and establishes a chemically defined platform for antifungal intervention and detection.

## Graphical abstract

A synthetic β‐mannan epitope enables both active and passive immunization against *Candida auris*. Conjugation to the carrier protein CRM_197_ induces protective antibody responses, while monoclonal antibodies targeting the same epitope support detection using a lateral flow assay, linking glycochemistry with antifungal prevention and diagnostics.

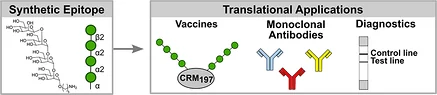

## 1 Introduction

*Candida auris* is an emerging multidrug‐resistant fungal pathogen responsible for invasive infections with high mortality rates and limited treatment options [1, 2, 3]. Since its first description in 2009, multiple genetically distinct clades have been identified, highlighting its rapid evolution and global spread [4, 5, 6]. Its persistence in healthcare settings, immune evasion, and resistance to antifungal drugs led the World Health Organization to classify *C. auris* as a critical priority pathogen [7]. Despite its clinical relevance, neither vaccines nor rapid, reliable diagnostics are currently available [7, 8, 9].

The fungal cell wall represents a key interface for immune recognition and consists of a complex network of polysaccharides, including β‐glucans, chitin, and mannans [10, 11, 12]. In *C. auris*, an expanded outer mannan layer shields underlying β‐glucans and contributes to immune evasion [13, 14, 15, 16, 17, 18, 19]. These mannans comprise an α‐(1,6)‐linked backbone decorated with α‐(1,2)‐, α‐(1,3)‐, β‐(1,2)‐, and β‐(1,6)‐linked mannose residues. Among these, terminal β‐(1,2)‐linked mannose residues represent immunologically relevant side‐chain motifs recognized by host antibodies and lectins [20, 21].

While antifungal immunity has long been attributed primarily to innate responses, increasing evidence highlights the importance of antibodies in controlling fungal infections [22, 23, 24, 25, 26, 27]. Glycan epitopes on fungal cell walls therefore offer defined molecular targets for vaccine design and antibody‐based detection [28, 29, 30]. However, natural fungal polysaccharides are structurally heterogeneous, complicating the identification of well‐defined immunogenic epitopes [30].

Synthetic glycans provide a chemically precise alternative that enables systematic investigation of structure‐function relationships in immune recognition. In this context, β‐(1,2)‐linked oligomannosides have emerged as promising epitopes for antibody binding and vaccine development [31, 32, 33].

Here, we show that a defined synthetic β‐mannan tetrasaccharide functions as a lead epitope against *C. auris*. Conjugation to the carrier protein CRM_197_ enables active immunization, while monoclonal antibodies raised against the same epitope support passive immunization and antibody‐based detection. Together, these results establish a chemically defined glycan epitope as a platform for antifungal intervention and diagnostic recognition.

## 2 Results and Discussion

### 2.1 Synthesis and Characterization of M13‐CRM_197_

The mannose tetrasaccharide **M13**, previously identified as a *Candida*‐related lead epitope [31, 32, 33], was synthesized following established protocols [32] and conjugated to the carrier protein CRM_197_ [34, 35] (Figure 1A). MALDI‐TOF mass spectrometry showed an increase in molecular weight from 59 138 to 71 513 Da, corresponding to an average loading of approximately 14 glycan units per protein (Figure 1B). SDS‐PAGE analysis confirmed successful conjugation and revealed heterogeneity in glycan loading (Figure 1C), consistent with steric and accessibility constraints reported for CRM_197_ conjugates [36, 37].

**FIGURE 1:**
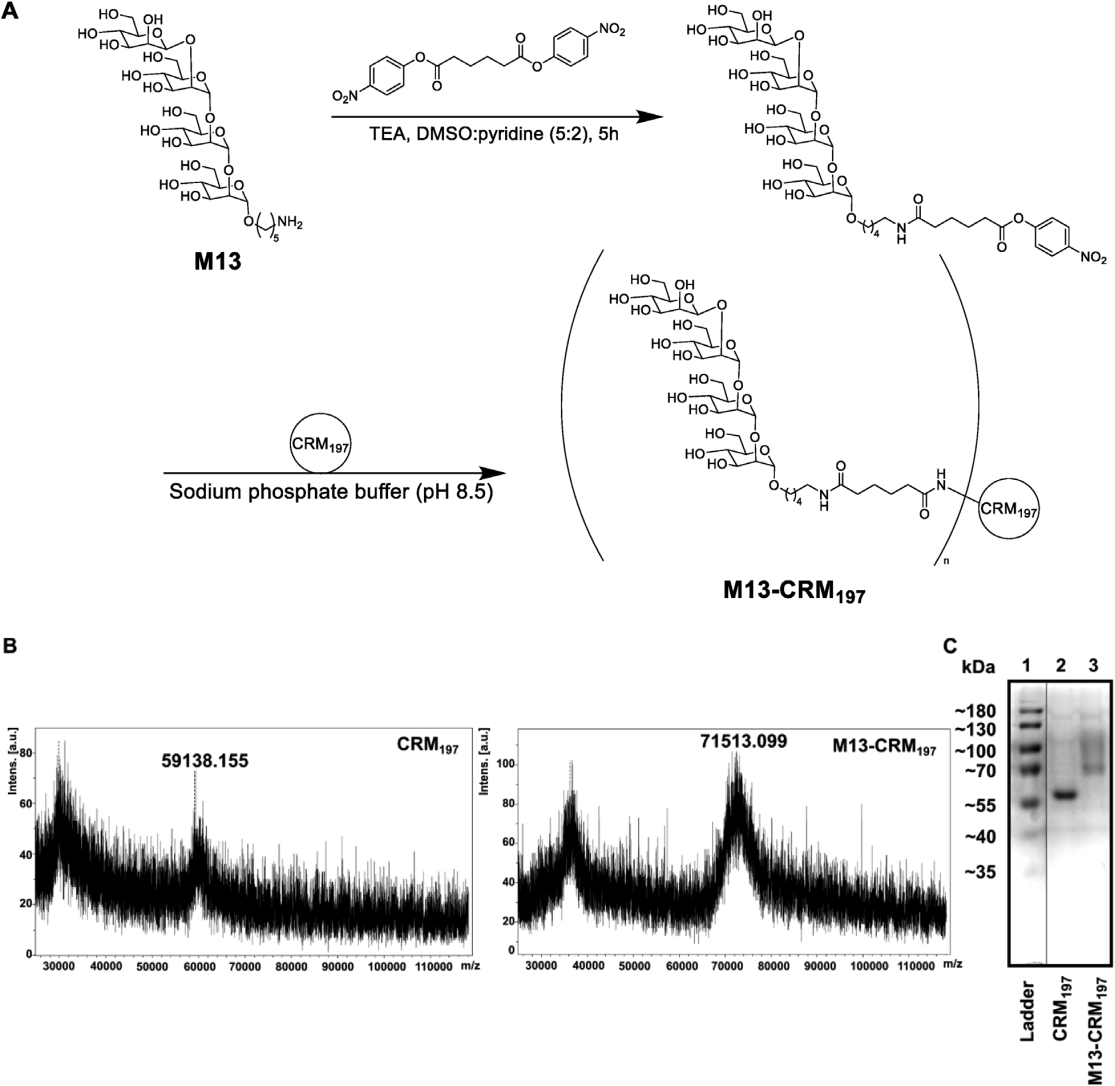
Synthesis and characterization of the glycoconjugate **M13**‐CRM_197_. (A) Schematic representation of the covalent conjugation of the synthetic β‐mannan tetrasaccharide **M13** to the carrier protein CRM_197_ to generate the glycoconjugate **M13**‐CRM_197_. (B) MALDI‐TOF mass spectra of CRM_197_ and **M13**‐CRM_197_ confirming successful glycan conjugation by the observed molecular weight shift. (C) SDS‐PAGE analysis of CRM_197_ (lane 2), **M13**‐CRM_197_ (lane 3), and a PageRuler Plus Prestained Protein Ladder (lane 1). Proteins were visualized by Coomassie Blue staining, confirming successful conjugation and heterogeneous glycan loading.

### 2.2 Epitope‐Specific Antibody Responses

Immunization with **M13**‐CRM_197_ elicited a robust humoral response (Figure 2A). Building on our previous identification of **M13** as the lead immunogenic β‐(1,2)‐mannan epitope of *C. auris* [33], glycan microarray analysis was used to verify the induction of **M13**‐specific antibodies following immunization and to evaluate their recognition of structurally related β‐(1,2)‐linked oligomannosides. Strong IgM responses were detected by Day 14, followed by the induction of IgG responses from Day 28 onward (Figure 2B,C and Dataset S1). Both IgM and IgG antibodies selectively recognized the **M13** epitope, with reduced binding to the trisaccharide **M12** and negligible binding to shorter glycans (Figure 2D and Dataset S1), demonstrating the importance of glycan length and β‐linkage for immune recognition.

**FIGURE 2:**
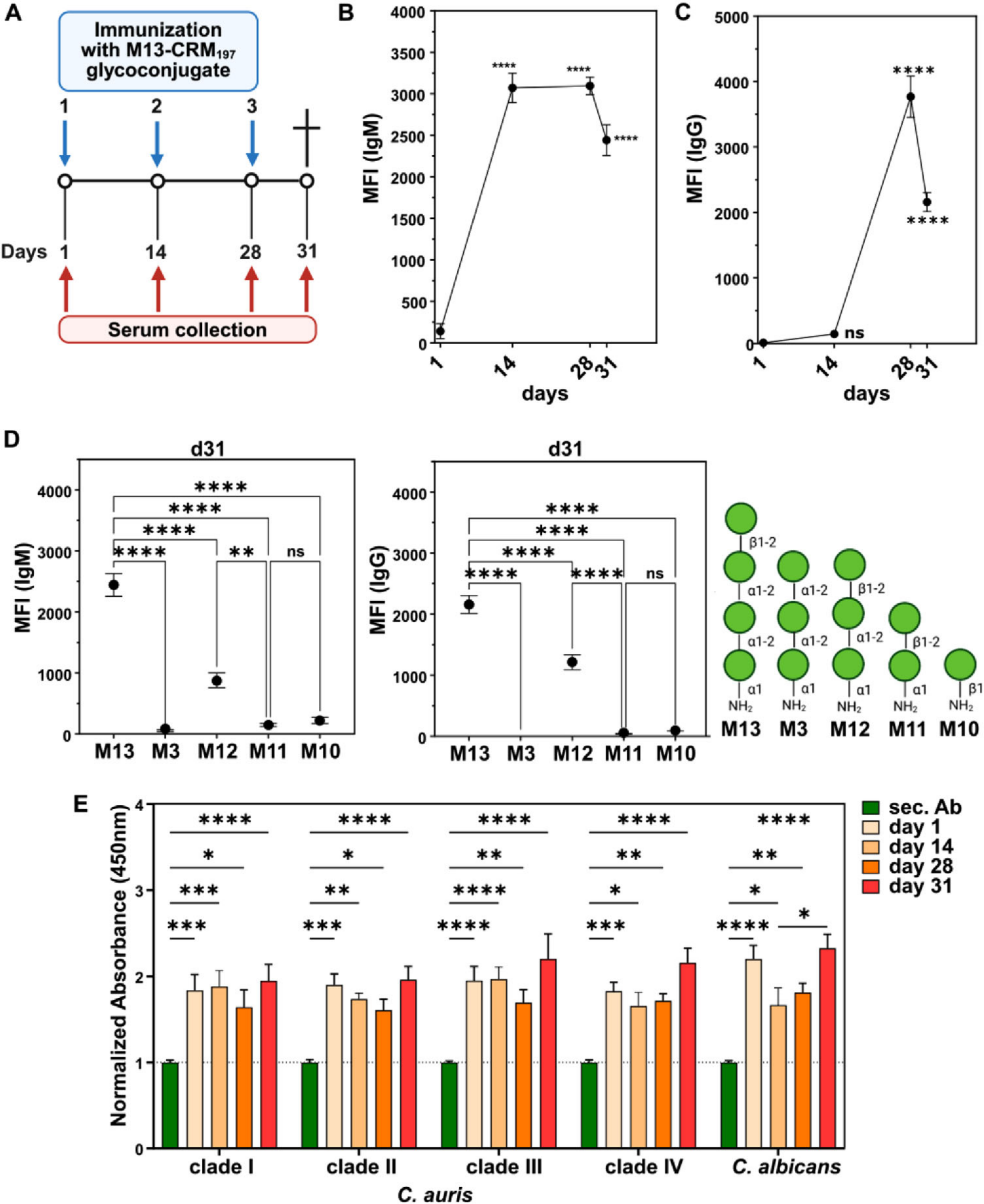
Epitope‐specific antibody responses induced by **M13**‐CRM_197_. (A) Immunization schedule of mice receiving the **M13**‐CRM_197_ glycoconjugate. (B, C) Glycan microarray analysis showing mean fluorescence intensity (MFI) of serum IgM (B) and IgG (C) antibodies specific for **M13** on Days 1, 14, 28, and 31 post‐immunization. (D) Epitope specificity of serum antibodies at Day 31 assessed by binding to the synthetic glycans **M13**, **M12**, **M11**, **M10**, and **M3**. (E) ELISA analysis of IgG antibody binding to fixed *C. auris* isolates from clades I–IV and *Candida albicans* WO‐1, normalized to the corresponding secondary antibody controls. Data represent mean ± SEM of four biological replicates (*n* = 4 female C57BL/6NRj mice). Statistical significance was determined by two‐way ANOVA with Tukey's multiple comparison test (B, C, E) or one‐way ANOVA (D); significance levels are indicated as follows: (****) *p* < 0.0001, (***) *p* < 0.0002, (**) *p* < 0.0021, and (*) *p* < 0.0332.

Pre‐immune sera showed IgG binding to *Candida* cells (Figure 2E and Dataset S2), consistent with natural cross‐reactive antibodies [33, 38, 39, 40], but no reactivity toward **M13** (Figure S1 and Dataset S1). **M13**‐specific responses emerged only after immunization, confirming the induction of antibodies directed against the defined synthetic glycan and distinguishing pre‐existing broad reactivity from vaccine‐induced, epitope‐specific antibody responses.

### 2.3 In Vivo Protection by Glycoconjugate Immunization

To evaluate functional relevance, immunized mice were challenged with *C. auris* (clade I) [41, 42] (Figure 3A). **M13**‐specific IgG responses were present at the time of infection (Figure S2 and Dataset S3). Immunized animals showed significantly reduced fungal burdens in kidneys and spleen compared to controls (Figure 3B,C and Dataset S4), demonstrating that the defined epitope confers protection in vivo.

**FIGURE 3:**
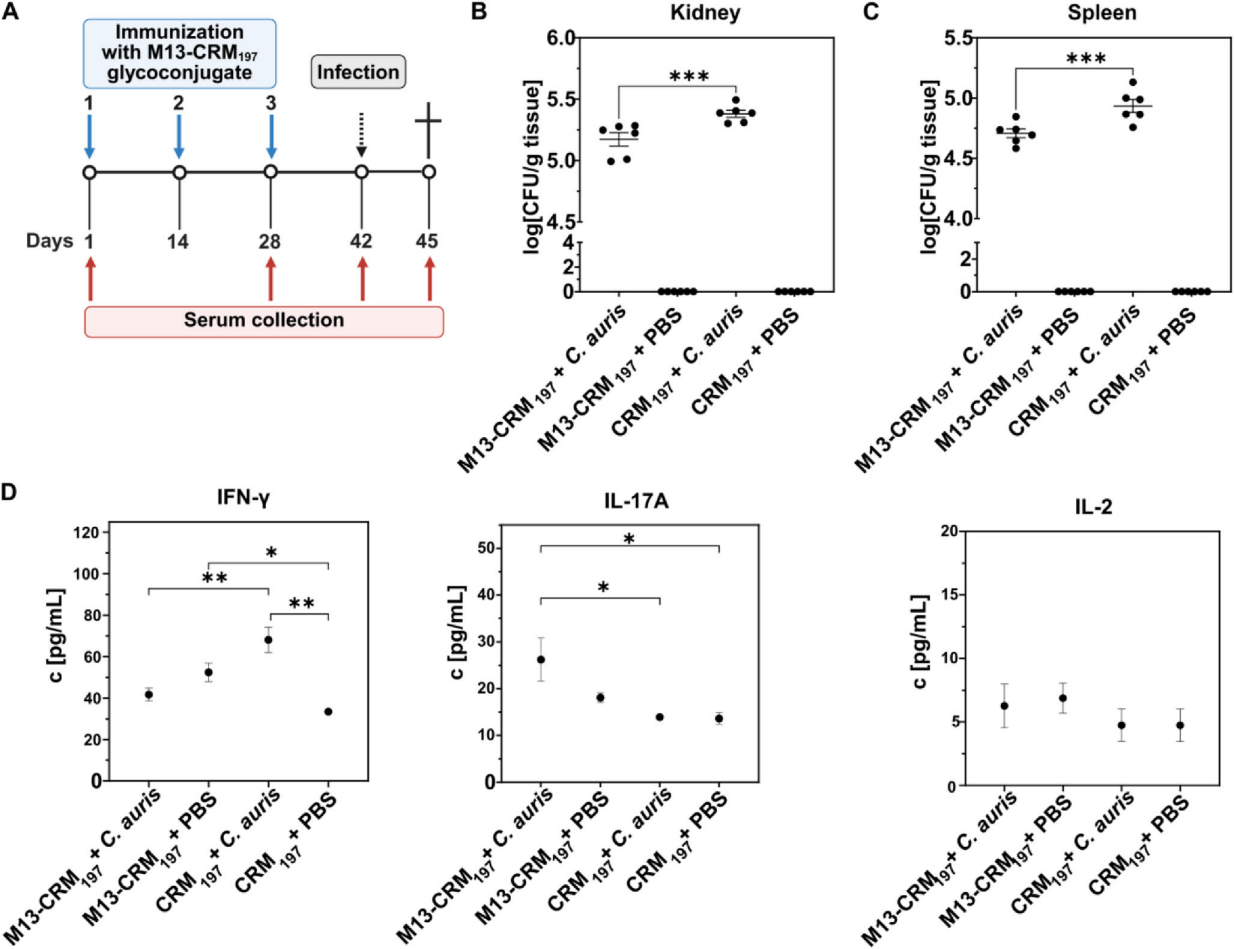
Protective efficacy of **M13**‐CRM_197_ immunization in a murine infection model. (A) Immunization and infection scheme (*n* = 6 mice per group). (B, C) Quantification of fungal burden in the kidneys (B) and spleens (C) at the study endpoint (Day 45 post‐infection). (D) Serum levels of IFN‐γ, IL‐17A, and IL‐2 measured in pooled serum samples collected at the study endpoint. Data represent mean ± SEM. Statistical significance was determined by one‐way ANOVA; significance levels are indicated as follows: (****) *p* < 0.0001, (***) *p* < 0.0002, (**) *p* < 0.0021, and (*) *p* < 0.0332.

Cytokine analysis revealed modulation of immune responses, with normalization of IFN‐γ and increased IL‐17A in immunized and infected animals (Figure 3D and Dataset S5), consistent with enhanced antifungal immunity [43, 44, 45, 46]. Correlation analysis showed an inverse relationship between IgG levels and fungal burden (Figure S3 and Datasets S3–S5), supporting a role for antibody‐mediated fungal control.

### 2.4 Generation and Characterization of Monoclonal Antibodies

Monoclonal antibodies recognizing the defined epitope were generated from immunized mice. Three IgG1 antibodies (7HF, 7KH, 7CG) were obtained (Figure 4A,B). To define the minimal β‐(1,2)‐mannan epitope recognized by the selected monoclonal antibodies, binding was assessed using a panel of structurally related synthetic oligomannosides for epitope mapping. Glycan microarray analysis demonstrated dose‐dependent binding to **M13** and differential recognition of shorter glycans (Figure 4C,D and Dataset S7). The microarray analysis was used to compare antibody recognition of structurally defined synthetic glycans and to define the minimal glycan motif recognized by the selected monoclonal antibodies. In contrast to the polyclonal serum response, which showed the strongest recognition of **M13**, the purified monoclonal antibodies exhibited maximal binding to **M12** while retaining binding to **M11**. This difference likely reflects the heterogeneous composition of the polyclonal antibody response compared with the single specificities of the monoclonal antibodies, together with differences in glycan presentation and accessibility on the microarray surface. All antibodies bound *Candida* cells in ELISA (Figure 4E and Dataset S8), while flow cytometry revealed distinct binding profiles across strains (Figure 4F and Dataset S9), reflecting differences in epitope presentation. Sequence analysis confirmed that the antibodies are clonally related but distinct (Figure S5).

**FIGURE 4:**
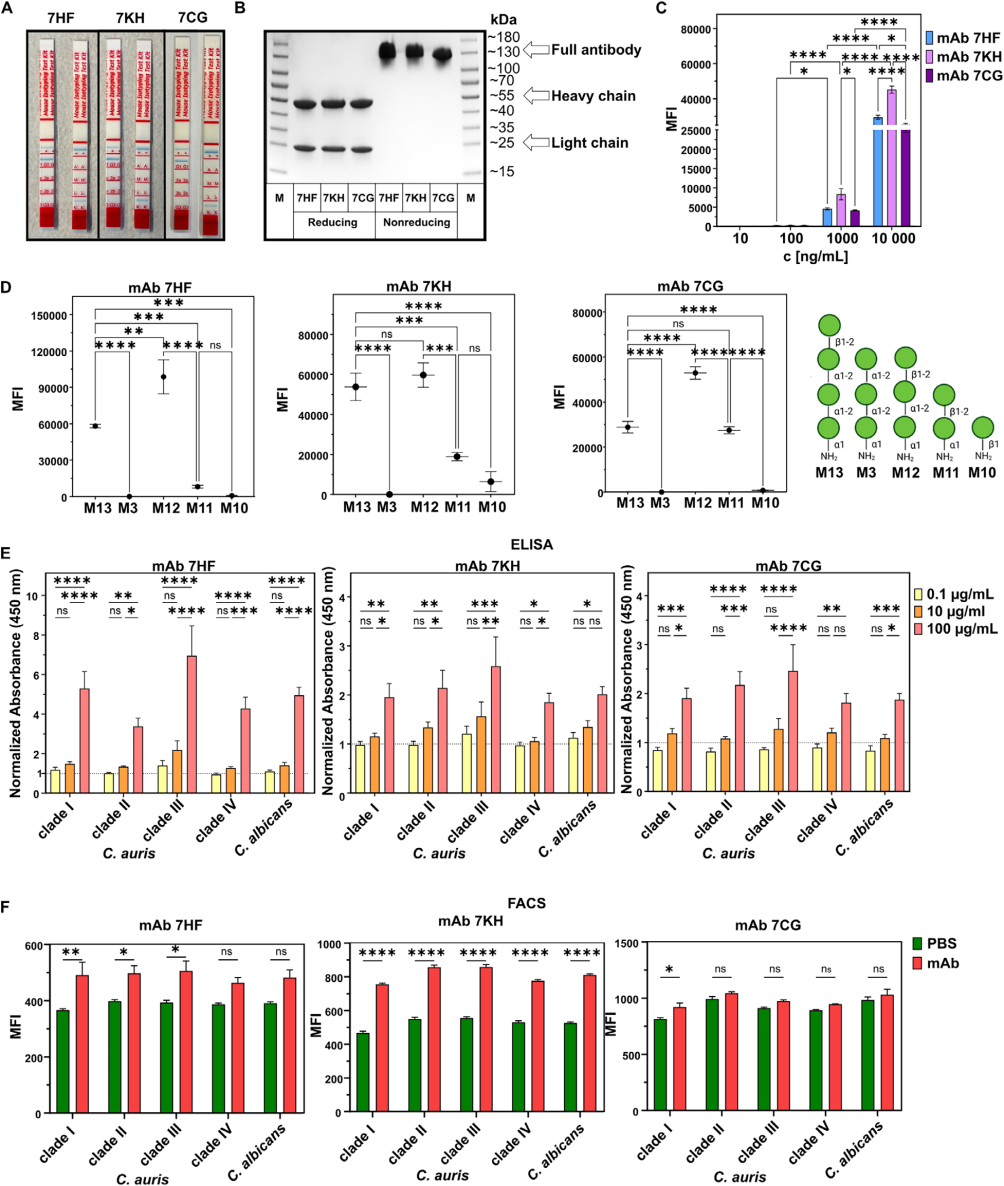
Characterization of monoclonal antibodies targeting the **M13** epitope. (A) Isotype determination of mAbs 7HF, 7KH, and 7CG using a mouse monoclonal antibody isotyping test kit, identifying all three as IgG1 antibodies with *κ* light chains. (B) SDS‐PAGE analysis under reducing and non‐reducing conditions confirming antibody purity and expected molecular weights. A PageRuler Plus Prestained Protein Ladder was used as the molecular weight marker, and proteins were visualized by Coomassie Blue staining. (C) Glycan microarray analysis showing mean fluorescence intensity (MFI) of antibody binding to the synthetic glycan **M13** across a concentration range (10–10,000 ng/mL). (D) Epitope specificity of the monoclonal antibodies (3000 ng/mL) assessed by binding to the synthetic glycans **M13**, **M12**, **M11**, **M10**, and **M3**, demonstrating differential recognition of structurally related β‐(1,2)‐linked oligomannosides. Note that the *y*‐axis scales differ between antibodies to facilitate visualization of the individual binding profiles. (E) ELISA analysis of antibody binding to fixed *C. auris* isolates from clades I–IV and *C. albicans* WO‐1 at concentrations of 0.1, 10, and 100 µg/mL. (F) Flow cytometry analysis of antibody binding (100 µg/mL) to the same panel of fixed *Candida* strains. Data represent mean ± SEM. Statistical significance was determined by two‐way ANOVA (C, E, and F) or one‐way ANOVA (D); significance levels are indicated as follows: (****) *p* < 0.0001, (***) *p* < 0.0002, (**) *p* < 0.0021, and (*) *p* < 0.0332.

### 2.5 Passive Immunization With Epitope‐Specific Antibodies

Passive immunization experiments revealed functional differences between antibodies (Figure 5A). Treatment with mAb 7HF significantly reduced fungal burden in the spleen but not in the kidney (Figure 5B,C and Dataset S10), indicating organ‐specific effects. In contrast, mAb 7KH did not confer protection and increased kidney fungal burden, possibly due to differences in Fc‐effector function, tissue distribution [47, 48, 49], or prozone‐like effects [50, 51].

**FIGURE 5:**
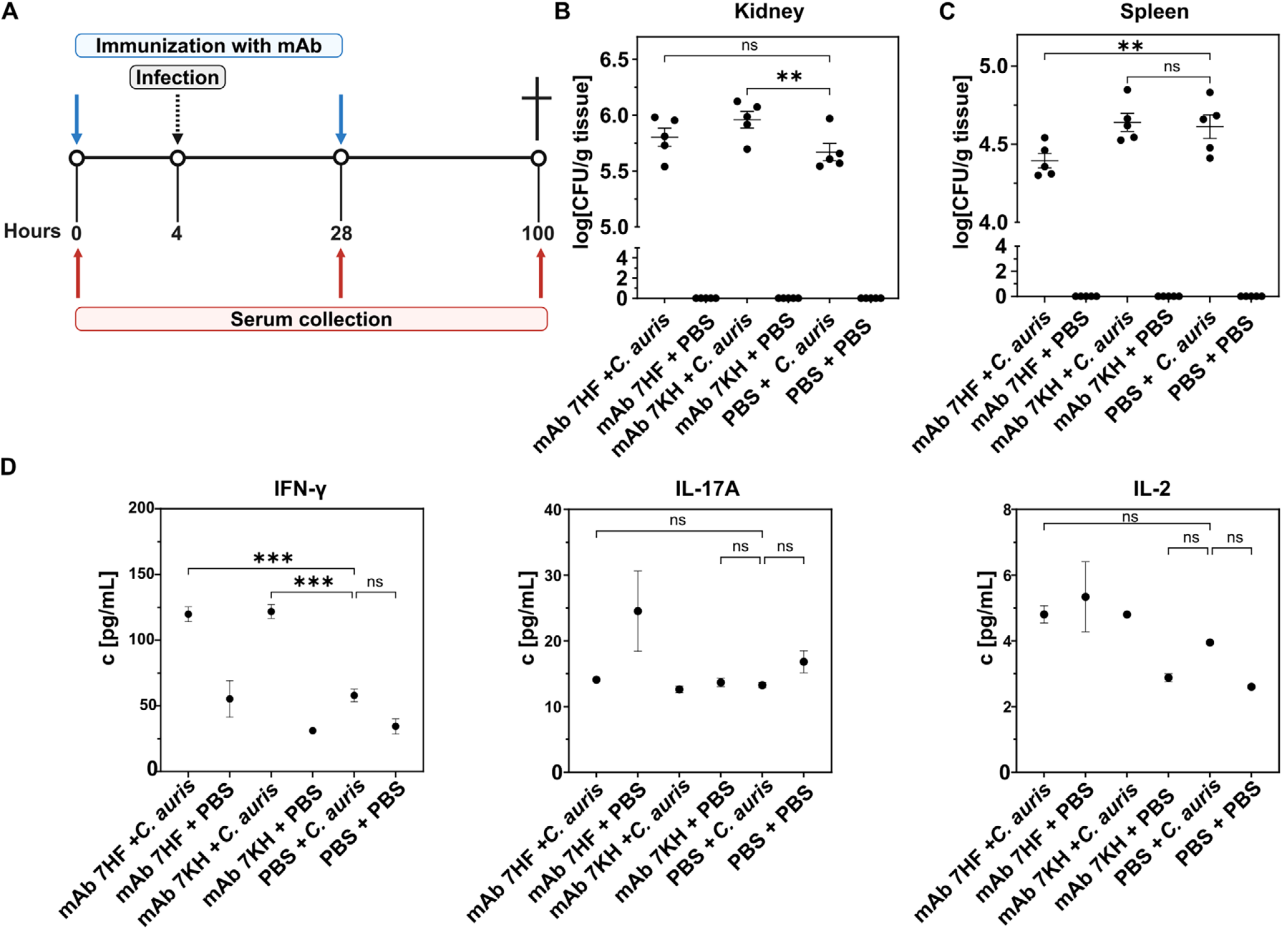
Passive immunization with monoclonal antibodies in a murine infection model. (A) Experimental design for passive immunization and *C. auris* infection (*n* = 5 mice per group). (B, C) Quantification of fungal burden in the kidneys (B) and spleens (C) at the study endpoint (100 h post‐infection). (D) Serum levels of IFN‐γ, IL‐17A, and IL‐2 measured in pooled serum samples collected at the study endpoint. Data represent mean ± SEM. Statistical significance was determined by one‐way ANOVA; significance levels are indicated as follows: (****) *p* < 0.0001, (***) *p* < 0.0002, (**) *p* < 0.0021, and (*) *p* < 0.0332.

Antibody treatment increased IFN‐γ levels compared to infection alone (Figure 5D and Dataset S11), suggesting enhanced immune activation, while IL‐17A and IL‐2 remained unchanged. These findings indicate that protective efficacy depends not only on binding but also on downstream immune mechanisms.

### 2.6 Antibody‐Based Detection by Lateral Flow Assay

The diagnostic potential of the epitope‐specific antibodies was evaluated using a lateral flow assay (Figure 6A). Based on its broad and consistent binding profile across the tested *Candida* strains, mAb 7KH was selected for proof‐of‐concept evaluation. mAb 7KH enabled detection of multiple *Candida* species and *C. auris* clades, with varying signal intensities (Figure 6B), likely reflecting differences in epitope exposure [15, 21, 52, 53]. Although not protective in vivo, 7KH showed robust binding and is suitable for diagnostic applications. The current assay does not distinguish between *C. auris* and *C. albicans*, and further optimization is required.

**FIGURE 6:**
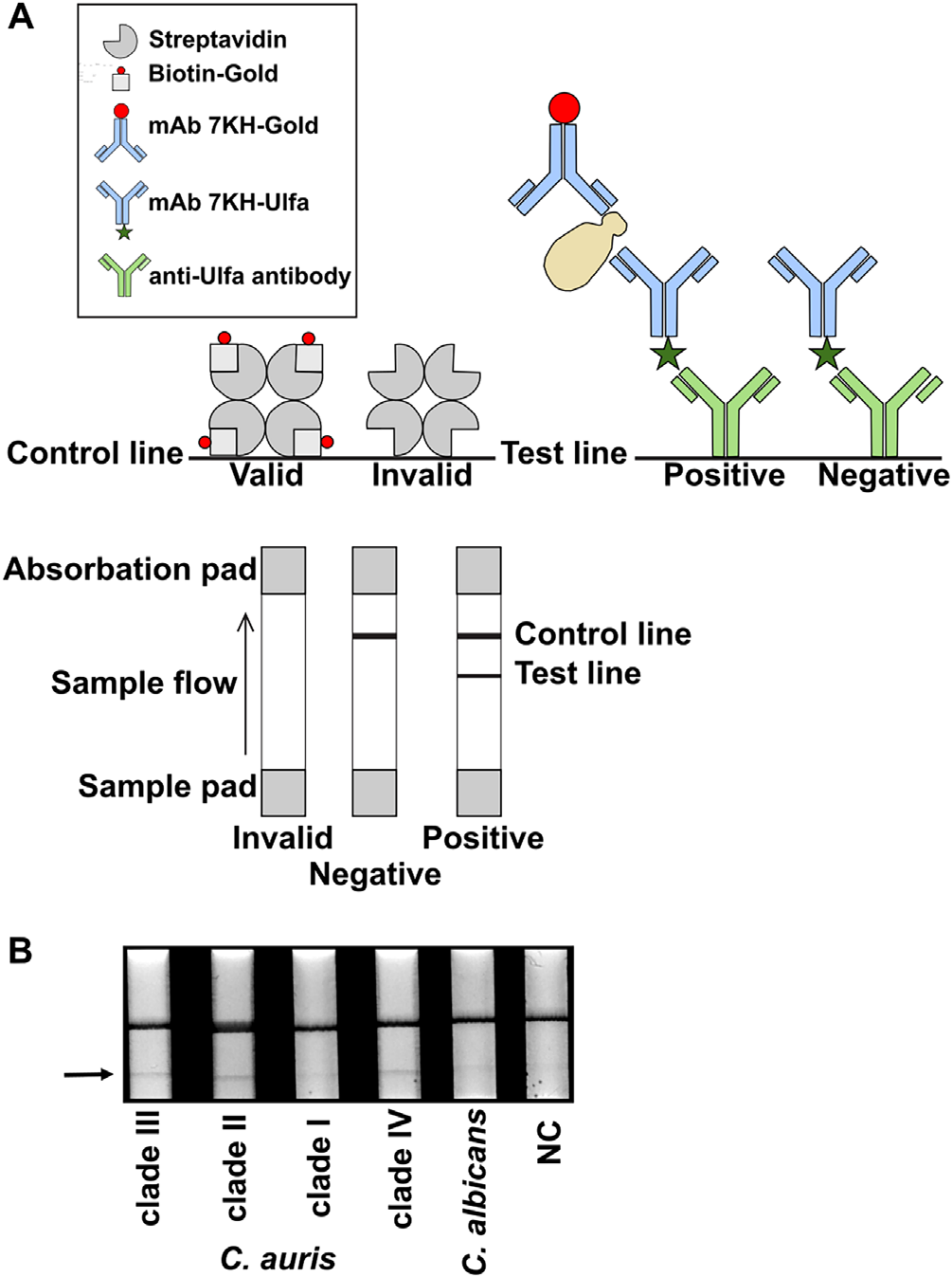
Antibody‐based detection of *Candida* species using a lateral flow assay. (A) Schematic representation of the sandwich‐format lateral flow assay using mAb 7KH as both capture and detection antibody. The test line contains immobilized anti‐Ulfa antibodies, while the control line contains immobilized streptavidin. (B) Detection of *C. auris* isolates from clades I–IV and *C. albicans* WO‐1 using the lateral flow assay. NC: negative control without *Candida* cells. The arrow indicates the position of the test line.

## 3 Conclusion

A single, chemically defined β‐mannan tetrasaccharide is sufficient for protective and diagnostic approaches against *C. auris*. Conjugation of the synthetic epitope to CRM_197_ enabled the induction of epitope‐specific humoral responses that translated into reduced fungal burden in vivo, highlighting the potential of structurally defined glycans for antifungal immunization. The selective recognition of M13 over shorter oligomannosides further emphasizes the importance of glycan length and β‐(1,2)‐linkage in determining immune specificity.

Monoclonal antibodies generated against the same synthetic epitope displayed distinct functional profiles, ranging from partial protection in passive immunization to robust binding suitable for diagnostic applications. These findings illustrate that binding affinity alone does not predict protective efficacy and underscore the role of Fc‐mediated mechanisms and tissue‐specific immune responses in determining functional outcomes.

The differential performance of the monoclonal antibodies further illustrates that epitope recognition alone is insufficient to predict functional activity, emphasizing the need to tailor antibodies for either therapeutic or diagnostic applications.

The same chemically defined synthetic β‐(1,2)‐mannan epitope thus served as the basis for active immunization, passive immunotherapy, and antibody‐based detection, demonstrating the versatility of a single glycan structure as a molecular platform for antifungal intervention. Importantly, the successful implementation of an antibody‐based lateral flow assay provides a proof of concept that a defined glycan epitope can be translated into a diagnostic format. β‐(1,2)‐Mannan epitopes are not unique to *C. auris* but have also been described in several clinically relevant *Candida* species, including *C. albicans*, *C. tropicalis*, *C. glabrata*, and *C. lusitaniae*. This broader distribution likely accounts for the observed cross‐reactivity of the generated antibodies [54]. While further optimization is required to improve sensitivity and species discrimination, this approach highlights the versatility of epitope‐specific antibodies derived from synthetic glycans.

The ability to translate precise glycan structures into both protective and analytical tools highlights chemically defined glycan epitopes as a versatile platform for antifungal immunization and diagnostic recognition. Future studies will focus on expanding epitope coverage, optimizing antibody functionality, and validating these approaches in clinically relevant settings.

## Author Contributions

**Emelie E. Reuber**: conceptualization, investigation, methodology, validation, visualization, formal analysis, data curation, writing – review and editing, and writing – original draft. **Arnab Pradhan**: conceptualization, investigation, methodology, writing – review and editing, project administration, and data curation. **Ian Leaves**: conceptualization, investigation, methodology, data curation, and writing – review and editing. **Rajat K. Singh**: writing – review and editing, methodology, conceptualization, and investigation. **Emer Hickey**: conceptualization, investigation, methodology, and writing – review and editing. **Carola Geiler**: investigation, methodology, and writing – review and editing. **Neil A. R. Gow**: supervision, resources, conceptualization, writing – review and editing, and funding acquisition. **Alistair J. P. Brown**: writing – review and editing, conceptualization, supervision, resources, and funding acquisition. **Peter H. Seeberger**: funding acquisition, conceptualization, writing – review and editing, resources, and supervision.

## Conflicts of Interest

The authors declare no conflicts of interest.

## Supporting information



The authors have cited additional references within the Supporting Information [31, 32, 34, 55, 56, 57, 58, 59].**Supporting File 1**: anie74122‐sup‐0001‐SuppMat.pdf.

**Supporting File 2**: anie74122‐sup‐0002‐Dataset.zip.

## Data Availability

The data that supports the findings of this study are available in the Supporting Information of this article.

## References

[1] S. Ahmad and W. Alfouzan , “ *Candida auris*: Epidemiology, Diagnosis, Pathogenesis, Antifungal Susceptibility, and Infection Control Measures to Combat the Spread of Infections in Healthcare Facilities,” Microorganisms 9 (2021): 807. 10.3390/microorganisms9040807 PMC8069182 33920482

[2] M. A. Pfaller and D. J. Diekema , “Epidemiology of Invasive Candidiasis: A Persistent Public Health Problem,” Clinical Microbiology Reviews 20 (2007): 133–163, 10.1128/CMR.00029-06. PMC1797637 17223626

[3] M. A. Pfaller , D. J. Diekema , D. L. Gibbs , et al., “Results From the Artemis Disk Global Antifungal Surveillance Study, 1997 to 2007: A 10.5‐Year Analysis of Susceptibilities of Candida Species to Fluconazole and Voriconazole as Determined by CLSI Standardized Disk Diffusion,” Journal of Clinical Microbiology 48 (2010): 1366–1377, 10.1128/JCM.02117-09. PMC2849609 20164282

[4] S. R. Lockhart , K. A. Etienne , S. Vallabhaneni , et al., “Simultaneous Emergence of Multidrug‐Resistant *Candida auris* on 3 Continents Confirmed by Whole‐Genome Sequencing and Epidemiological Analyses,” Clinical Infectious Diseases 64 (2017): 134–140, 10.1093/cid/ciw691. PMC5215215 27988485

[5] B. Spruijtenburg , H. Badali , M. Abastabar , et al., “Confirmation of Fifth *Candida auris* Clade by Whole Genome Sequencing,” Emerging Microbes & Infections 11 (2022): 2405–2411. 10.1080/22221751.2022.2125349 PMC9586689 36154919

[6] C. Suphavilai , K. K. K. Ko , K. M. Lim , et al., “Detection and Characterisation of a Sixth *Candida auris* Clade in Singapore: A Genomic and Phenotypic Study,” The Lancet Microbe 5 (2024): 100878, 10.1016/S2666-5247(24)00101-0. 39008997

[7] WHO Fungal Priority Pathogens List to Guide Research, Development and Public Health Action 1st ed. (World Health Organization, 2022).

[8] M. Morrell , V. J. Fraser , and M. H. Kollef , “Delaying the Empiric Treatment of Candida Bloodstream Infection Until Positive Blood Culture Results Are Obtained: A Potential Risk Factor for Hospital Mortality,” Antimicrobial Agents and Chemotherapy 49 (2005): 3640–3645, 10.1128/AAC.49.9.3640-3645.2005. PMC1195428 16127033

[9] I. Pitua and M. Okello‐Obol , “Urgent Need for Scaling up Vaccine Research on WHO Priority Fungal Pathogens,” EBioMedicine 113 (2025): 105583, 10.1016/j.ebiom.2025.105583. PMC11959649 39890506

[10] N. A. R. Gow , J. P. Latge , and C. A. Munro , “The Fungal Cell Wall: Structure, Biosynthesis, and Function,” Microbiology Spectrum 5 (2017): 10–1128. 10.1128/microbiolspec.funk-0035-2016 PMC11687499 28513415

[11] N. A. R. Gow and M. D. Lenardon , “Architecture of the Dynamic Fungal Cell Wall,” Nature Reviews Microbiology 21 (2023): 248–259, 10.1038/s41579-022-00796-9. 36266346

[12] C. Bekirian , I. Valsecchi , S. Bachellier‐Bassi , et al., “β‐1,6‐Glucan Plays a central Role in the Structure and Remodeling of the Bilaminate Fungal Cell Wall,” Elife 13 (2024): RP100569. 10.7554/eLife.100569 PMC11620752 39636210

[13] B. Yadav , H. M. Mora‐Montes , J. Wagener , et al., “Differences in Fungal Immune Recognition by Monocytes and Macrophages: N‐Mannan Can be a Shield or Activator of Immune Recognition,” Cell Surface 6 (2020): 100042, 10.1016/j.tcsw.2020.100042. PMC7750734 33364531

[14] M. S. Graus , M. J. Wester , D. W. Lowman , et al., “Mannan Molecular Substructures Control Nanoscale Glucan Exposure in Candida,” Cell Reports 24 (2018): 2432–2442, 10.1016/j.celrep.2018.07.088. PMC6204226 30157435

[15] S. Dakalbab , R. Hamdy , P. Holigova , et al., “Uniqueness of *Candida auris* Cell Wall in Morphogenesis, Virulence, Resistance, and Immune Evasion,” Microbiological Research 286 (2024): 127797, 10.1016/j.micres.2024.127797. 38851008

[16] X. Huang , Y. Liu , T. Ni , et al., “g, a Potent Antifungal Candidate, Enhances *Candida albicans* Immunogenicity by Unmasking β‐Glucan in Fungal Cell Wall,” Frontiers in Microbiology 11 (2020): 1324, 10.3389/fmicb.2020.01324. PMC7338940 32695076

[17] Y. Wang , Y. Zou , X. Chen , et al., “Innate Immune Responses Against the Fungal Pathogen *Candida auris* ,” Nature Communications 13 (2022): 3553, 10.1038/s41467-022-31201-x. PMC9213489 35729111

[18] S. M. G. Selisana , X. Chen , E. Mahfudhoh , et al., “Alteration of β‐Glucan in the Emerging Fungal Pathogen *Candida auris* Leads to Immune Evasion and Increased Virulence,” Medical Microbiology and Immunology 213 (2024): 13, 10.1007/s00430-024-00795-y. PMC11226559 38967888

[19] A. Pradhan , O. A. Nev , I. Leaves , et al., “Protein Kinase A Signaling Regulates Immune Evasion by Shaving and Concealing Fungal β‐1,3‐Glucan,” PNAS 122 (2025): e2423864122, 10.1073/pnas.2423864122. PMC12184398 40489619

[20] L. Yan , K. Xia , Y. Yu , et al., “Unique Cell Surface Mannan of Yeast Pathogen *Candida auris* With Selective Binding to IgG,” ACS Infectious Diseases 6 (2020): 1018–1031, 10.1021/acsinfecdis.9b00450. 32233507

[21] M. Bruno , S. Kersten , J. M. Bain , et al., “Transcriptional and Functional Insights Into the Host Immune Response Against the Emerging Fungal Pathogen *Candida auris* ,” Nature Microbiology 5 (2020): 1516–1531, 10.1038/s41564-020-0780-3. PMC9204844 32839538

[22] F. De Bernardis , M. Boccanera , D. Adriani , E. Spreghini , G. Santoni , and A. Cassone , “Protective Role of Antimannan and Anti‐Aspartyl Proteinase Antibodies in an Experimental Model of *Candida albicans* Vaginitis in Rats,” Infection and Immunity 65 (1997): 3399–3405. 10.1128/iai.65.8.3399-3405.1997 PMC175481 9234804

[23] Y. Han , M. A. Ulrich , and J. E. Cutler , “ *Candida albicans* Mannan Extract–Protein Conjugates Induce a Protective Immune Response Against Experimental Candidiasis,” Journal of Infectious Diseases 179 (1999): 1477–1484, 10.1086/314779. 10228070

[24] M. Wich , S. Greim , M. Ferreira‐Gomes , et al., “Functionality of the human Antibody Response to *Candida albicans* ,” Virulence 12 (2021): 3137–3148, 10.1080/21505594.2021.2015116. PMC8923069 34923920

[25] A. Torosantucci , P. Chiani , C. Bromuro , et al., “Protection by Anti‐β‐Glucan Antibodies Is Associated With Restricted β‐1,3 Glucan Binding Specificity and Inhibition of Fungal Growth and Adherence,” PLoS One 4 (2009): e5392. 10.1371/journal.pone.0005392 PMC2670538 19399183

[26] S. Singh , S. Nabeela , A. Barbarino , A. S. Ibrahim , and P. Uppuluri , “Antibodies Targeting *Candida albicans* Als3 and Hyr1 Antigens Protect Neonatal Mice From Candidiasis,” Frontiers in Immunology 13 (2022): 925821, 10.3389/fimmu.2022.925821. PMC9355692 35935947

[27] P. Uppuluri , S. Singh , A. Alqarihi , et al., “Human Anti‐Als3p Antibodies Are Surrogate Markers of NDV‐3A Vaccine Efficacy Against Recurrent Vulvovaginal Candidiasis,” Frontiers in Immunology 9 (2018): 1349, 10.3389/fimmu.2018.01349. PMC6013566 29963049

[28] B. Pathakumari , G. Liang , and W. Liu , “Immune Defence to Invasive Fungal Infections: A Comprehensive Review,” Biomedicine & Pharmacotherapy 130 (2020): 110550, 10.1016/j.biopha.2020.110550. 32739740

[29] M. Shukla , P. Chandley , and S. Rohatgi , “The Role of B‐Cells and Antibodies Against Candida Vaccine Antigens in Invasive Candidiasis,” Vaccines 9 (2021): 1159. 10.3390/vaccines9101159 PMC8540628 34696267

[30] M. Mikulska , T. Calandra , M. Sanguinetti , D. Poulain , and C. Viscoli , “The Use of Mannan Antigen and Anti‐Mannan Antibodies in the Diagnosis of Invasive Candidiasis: Recommendations From the Third European Conference on Infections in Leukemia,” Third European Conference on Infections in Leukemia 14 (2010): R222. 10.1186/cc9365 PMC3219989 21143834

[31] R. K. Singh , *Chemical Synthesis of Oligosaccharides as Basis for the Development of Carbohydrate‐Based Vaccine Candidates Against Streptococcus Suis Serotype 18 and* Candida auris (Freie Universitaet Berlin Germany, 2023).

[32] R. K. Singh , E. E. Reuber , M. Bruno , M. G. Netea , and P. H. Seeberger , “Synthesis of Oligosaccharides to Identify an Immunologically Active Epitope Against *Candida auris* Infection,” Chemical Science 14 (2023): 7559–7563, 10.1039/D3SC01242E. PMC10337753 37449061

[33] E. E. Reuber , E. Hickey , A. Pradhan , et al., “Glycan Microarray Analysis of Candida‐Related Antibodies in human and Mice Sera Guides Biomarker Discovery and Vaccine Development,” PNAS 122 (2025): e2505340122, 10.1073/pnas.2505340122. PMC12501125 40996794

[34] M. P. Lisboa , N. Khan , C. Martin , et al., “Semisynthetic Glycoconjugate Vaccine Candidate Against Streptococcus pneumoniae Serotype 5,” PNAS 114 (2017): 11063–11068, 10.1073/pnas.1706875114. PMC5651752 28973947

[35] S. G. Parameswarappa , K. Reppe , A. Geissner , et al., “A Semi‐Synthetic Oligosaccharide Conjugate Vaccine Candidate Confers Protection Against Streptococcus pneumoniae Serotype 3 Infection,” Cell Chemical Biology 23 (2016): 1407–1416, 10.1016/j.chembiol.2016.09.016. PMC5234679 27818299

[36] S. Crotti , H. Zhai , J. Zhou , et al., “Defined Conjugation of Glycans to the Lysines of CRM 197 Guided by Their Reactivity Mapping,” ChemBioChem 15 (2014): 836–843, 10.1002/cbic.201300785. 24616190

[37] U. Moginger , A. Resemann , C. E. Martin , et al., “Cross Reactive Material 197 Glycoconjugate Vaccines Contain Privileged Conjugation Sites,” Scientific Reports 6 (2016): 20488, 10.1038/srep20488. PMC4740906 26841683

[38] D. M. Klinman , “Cross‐Reactivity of IgG and IgM Secreting B Cells in Normal and Autoimmune Mice,” Journal of Autoimmunity 7 (1994): 1–11, 10.1006/jaut.1994.1001. 8198696

[39] M. Vilanova , L. Teixeira , I. Caramalho , et al., “Protection Against Systemic Candidiasis in Mice Immunized With Secreted Aspartic Proteinase 2,” Immunology 111 (2004): 334–342, 10.1111/j.1365-2567.2004.01819.x. PMC1782415 15009435

[40] M. Swidergall and S. LeibundGut‐Landmann , “Immunosurveillance of *Candida albicans* Commensalism by the Adaptive Immune System,” Mucosal Immunology 15 (2022): 829–836, 10.1038/s41385-022-00536-5. PMC9385492 35778599

[41] D. Kappel , H. Gifford , A. Brackin , et al., “Genomic Epidemiology Describes Introduction and Outbreaks of Antifungal Drug‐Resistant *Candida auris* ,” NPJ Antimicrob Resist 2 (2024): 26, 10.1038/s44259-024-00043-6. PMC11442302 39359891

[42] J. S. Kim , H. Cha , and Y. S. Bahn , “Comprehensive Overview of *Candida auris*: An Emerging Multidrug‐Resistant Fungal Pathogen,” Journal of Microbiology and Biotechnology 34 (2024): 1365–1375, 10.4014/jmb.2404.04040. PMC11294645 38881183

[43] D. Gozalbo , V. Maneu , and M. L. Gil , “Role of IFN‐Gamma in Immune Responses to *Candida albicans* Infections,” Frontiers in Bioscience‐Landmark 19 (2014): 1279, 10.2741/4281. 24896350

[44] F. L. van de Veerdonk and M. G. Netea , “T‐Cell Subsets and Antifungal Host Defenses,” Current Fungal Infection Reports 4 (2010): 238–243. 10.1007/s12281-010-0034-6 PMC2949562 20976286

[45] S. L. Gaffen , N. Hernandez‐Santos , and A. C. Peterson , “IL‐17 Signaling in Host Defense Against *Candida albicans* ,” Immunologic Research 50 (2011): 181–187, 10.1007/s12026-011-8226-x. PMC3257840 21717069

[46] H. R. Conti , F. Shen , N. Nayyar , et al., “Th17 cells and IL‐17 Receptor Signaling Are Essential for Mucosal Host Defense Against Oral Candidiasis,” Journal of Experimental Medicine 206 (2009): 299–311, 10.1084/jem.20081463. PMC2646568 19204111

[47] R. B. Ashman , C. S. Farah , S. Wanasaengsakul , Y. Hu , G. Pang , and R. L. Clancy , “Innate versus Adaptive Immunity in *Candida albicans* Infection,” Immunology and Cell Biology 82 (2004): 196–204, 10.1046/j.0818-9641.2004.01217.x. 15061774

[48] D. Leipold and S. Prabhu , “Pharmacokinetic and Pharmacodynamic Considerations in the Design of Therapeutic Antibodies,” Clinical and Translational Science 12 (2019): 130–139, 10.1111/cts.12597. PMC6440574 30414357

[49] V. A. Thomas and J. P. Balthasar , “Understanding Inter‐Individual Variability in Monoclonal Antibody Disposition,” Antibodies 8 (2019): 56. 10.3390/antib8040056 PMC6963779 31817205

[50] M. J. Sevilla , B. Robledo , A. Rementeria , M. D. Moragues , and J. Ponton , “A Fungicidal Monoclonal Antibody Protects Against Murine Invasive Candidiasis,” Infection and Immunity 74 (2006): 3042–3045, 10.1128/IAI.74.5.3042-3045.2006. PMC1459740 16622248

[51] T. R. Kozel , R. S. MacGill , A. Percival , and Q. Zhou , “Biological Activities of Naturally Occurring Antibodies Reactive With *Candida albicans* Mannan,” Infection and Immunity 72 (2004): 209–218, 10.1128/IAI.72.1.209-218.2004. PMC343987 14688098

[52] M. V. Horton , C. J. Johnson , R. Zarnowski , et al., “Candida auris Cell Wall Mannosylation Contributes to Neutrophil Evasion Through Pathways Divergent From Candida albicans and Candida glabrata,” mSphere 6 (2021): e0040621, 10.1128/mSphere.00406-21. PMC8265655 34160238

[53] I. A. Banda‐Flores , D. Torres‐Tirado , H. M. Mora‐Montes , G. Perez‐Flores , and L. A. Perez‐Garcia , “Resilience in Resistance: The Role of Cell Wall Integrity in Multidrug‐Resistant Candida,” Journal of Fungi 11 (2025): 271. 10.3390/jof11040271 PMC12028102 40278091

[54] N. Shibata , H. Kobayashi , and S. Suzuki , “Immunochemistry of Pathogenic Yeast, *Candida* Species, Focusing on Mannan,” Proceedings of the Japan Academy, Ser B, Physical and Biological Sciences 88 (2012): 250–265, 10.2183/pjab.88.250. PMC3410142 22728440

[55] P. N. Papapanou , M. Sanz , N. Buduneli , et al., “Periodontitis: Consensus Report of Workgroup 2 of the 2017 World Workshop on the Classification of Periodontal and Peri‐Implant Diseases and Conditions,” Journal of Periodontology 89 (2018): S173–S182. 10.1002/JPER.17-0721 29926951

[56] L. Meyer , T. Lopez , R. Espinosa , C. F. Arias , C. Vollmers , and R. M. DuBois , “A Simplified Workflow for Monoclonal Antibody Sequencing,” PLoS One 14 (2019): e0218717, 10.1371/journal.pone.0218717. PMC6590890 31233538

[57] V. Giudicelli , X. Brochet , and M. P. Lefranc , “IMGT/V‐Quest: IMGT Standardized Analysis of the Immunoglobulin (IG) and T Cell Receptor (TR) Nucleotide Sequences,” Cold Spring Harbor Protocols 2011 (2011): 695–715. 10.1101/pdb.prot5633 21632778

[58] S. Palliyil , M. Mawer , S. A. Alawfi , et al., “Monoclonal Antibodies Targeting Surface‐Exposed Epitopes of *Candida albicans* Cell Wall Proteins Confer in Vivo Protection in an Infection Model,” Antimicrobial Agents and Chemotherapy 66 (2022): e0195721, 10.1128/aac.01957-21. PMC9017365 35285676

[59] E. G. Youssef , L. Zhang , S. Alkhazraji , et al., “Monoclonal IgM Antibodies Targeting *Candida albicans* Hyr1 Provide Cross‐Kingdom Protection Against Gram‐Negative Bacteria,” Frontiers in Immunology 11 (2020): 76, 10.3389/fimmu.2020.00076. PMC7045048 32153560

[60] E. E. Reuber , “Synthetic Glycans in Infectious Disease: From Epitope Discovery to Diagnostic and Vaccine Development” (Freie Universität, 2025), 10.17169/refubium-51328.

