# Supplementary material for: A Synthetic β‐Mannan Epitope Enables Immunization and Detection of Candida auris
Source: Angew Chem Int Ed Engl. 2026 Aug 11;65(41):e9466991. doi: 10.1002/anie.9466991 (PMC13638085; doi:10.1002/anie.9466991)
Supplement: Supplementary file 1 [file ANIE-65-e9466991-s001.pdf]

A Synthetic β-Mannan Epitope Enables Immunization and Detection of *Candida auris*

Emelie E. Reuber,[a,b] Arnab Pradhan,[c] Ian Leaves,[c] Rajat K. Singh,[a,b] Emer Hickey,[c] Carola Geiler,[b] Neil A. R. Gow,[c] Alistair J. P. Brown,[c] Peter H. Seeberger[a,b]*

[a] E. E. Reuber, R. K. Singh, P. H. Seeberger
Department of Chemistry and Biochemistry, Freie Universität Berlin
14195 Berlin, Germany
E-mail: peter.seeberger@mpikg.mpg.de

[b] E. E. Reuber, R. K. Singh, C. Geiler, P. H. Seeberger
Department of Biomolecular Systems, Max Planck Institute of Colloids and Interfaces
14476 Potsdam, Germany

[c] A. Pradhan, I. Leaves, E. Hickey, N. A. R. Gow, A. J. P. Brown
MRC Centre for Medical Mycology, University of Exeter
Exeter EX4 4QD, UK

Supplementary Information

Material and Methods

Ethical statement

The glycoconjugate immunization experiment was approved by Landesamt für Gesundheit und Soziales Berlin, Germany (Approval ID: G 0329/18) and the mice were housed at the Max Planck Institute for Infection Biology, Berlin, Germany in groups of four animals in individually ventilated cages (Tecniplast, Hohenpeißenberg, Germany) under specific pathogen-free conditions. The experiment was carried out in accordance with institutional guidelines and with the regulations of the Federation of European Laboratory Animal Science Associations (FELASA). Animal experiments performed at the University of Exeter (establishment license number X7C5DF140) were approved by the University of Exeter Ethical Review Committee and were compliant with the United Kingdom Home Office licenses for research on animals (project license number PP9965358). BALB/c mice from Charles River were housed under specific pathogen-free conditions. Female mice were used in order to minimize variations associated with gender-specific immune responses and to ensure consistency with previous immunological studies. The fungal inoculum was selected based on preliminary, non-lethal pilot studies to ensure detectable fungal burden without causing severe illness.

Candida strains and growth conditions

C. auris 1747 NCPF8980 (clade III), *C. auris* 1748 NCPF8984 (clade II), *C. auris* 1750 NCPF8985 (clade I), *C. auris* 1762 B11244 (clade IV) and *C. albicans* WO-1 (mating type alpha) were cultured in YPD broth (Sigma-Aldrich, Cat. No Y1375) or plated on YPD agar (Sigma-Aldrich, Cat. No Y1500) plates at 30°C and 5% CO_2. NCPF strains were provided by the Health Protection Agency at Bristol, UK. WO-1 was a gift from D. Soll, Univ. Iowa, USA. In Exeter, for animal studies, *C. auris* cells were grown overnight in YPD at 30°C, 200 rpm, without CO_2 supplementation.

Glycan synthesis

Candida-related glycans were synthesized following established protocols.[1, 2]

Preparation of glycoconjugate

The synthetic mannose-tetrasaccharide **M13** was conjugated to the carrier protein CRM197 using a protocol described previously.[3, 4] Saccharide **M13** (1.03 mg) was dissolved in 200 µL DMSO, 25 µL pyridine and 10 µL triethylamine were added. Ten equivalents (5.3 mg, 13.7 µmol) of (4-nitrophenyl) adipate linker in dimethylsulfoxide were added to the saccharide solution. After 5 h of stirring, the reaction mixture was frozen in liquid nitrogen and subsequently lyophilized to give a crude white solid. The residue was washed with chloroform (5x 100 µL) and dichloromethane (3x 100 µL) to remove excess linker. CRM_{197} (1 mg, Pfénex, Cat. No 3452H002) was transferred into a 10kDa MWCO Amicon® Ultra centrifugal filter (Merck Millipore, Cat. No UFC5010) and washed with autoclaved water (3×350 µL) and phosphate buffer 0.1 M pH 8.0 (3×350 µL). The concentrated CRM_{197} was transferred into the vial containing the **M13** PNP ester and the reaction was stirred slowly for 24 h at room temperature. The solution was transferred onto a centrifugal filter and washed with phosphate buffer 0.1 M pH 8.0 (3×350 µL) and autoclaved water (3×350 µL). Before washing the mixture with phosphate buffered saline (2×350 µL), a 5 µL sample was taken for analysis. The conjugate was stored at -20°C. The average molecular size of the conjugate was characterized by matrix-assisted laser desorption/ionization (MALDI) analysis using 2,4-dihydroxyacetophenone (DHAP) as matrix with an Autoflex Speed MALDI-TOF system (Bruker Daltonics; Bremen, Germany) and the data was analyzed with the FlexAnalysis software provided with the instrument. Glycoconjugate (1 µg) and CRM197 were dissolved in SDS-PAGE loading buffer (250 mM Tris (pH 6.8), 5 % (v/v) beta-mercaptoethanol, 10 % (w/v) SDS, 0.4% Bromophenol Blue, 50 % (v/v) glycerol) and were resolved on a 10% SDS-PAGE gel. The electrophoresis was carried out at 120 V and 25 mA for 90 min in the electrode buffer and stained with 0.5% (w/v) Coomassie Brilliant Blue R-250 for 30 min and revealed in an Imager instrument (Gel DocTM EZ Imager, Bio-Rad).

Glycoconjugate vaccine formulation

A total of 1 µg **M13** conjugated to CRM_{197} or, in the case of the negative control, PBS, was mixed 1:1 with Alhydrogel® adjuvant 2% (InvivoGen, Cat. Code vac-alu-50) and phosphate buffer (PBS) was added to a total volume of 100 µl per mouse. To adsorb the antigen to the alum matrix, the mixture was incubated overnight at 4 °C under rotation.

Mice immunization with glycoconjugate

Four, eight week old C57BL/6NRj female mice (Janvier, Le 10 Genest-Saint Isle, France) were immunized subcutaneously (s.c.) with 0.1 mL of 1 µg/mouse per immunization. The animals were immunized on day 1 (primary immunization) and boosted on days 14 and 28. Blood was collected at day 1 (pre-immune), 14, 28 and 31 (terminal bleed). Sera were stored at -20°C. Spleens were harvested for the generation of hybridoma.

Glycan microarray

The glycans were dissolved at 0.1 mM in 50 mM sodium phosphate buffer pH 8.5 and printed in 64 identical fields to NEXTERION® 3-D Hydrogel coated glass slides (Schott) using a non-contact sciFLEXARRAYER S12 microarray spotter (Scienion, Berlin, Germany). After incubation overnight in a humidified box, the remaining NHS groups of the slides were quenched with ethanolamine. The slides were blocked with 1% (w/v) bovine serum albumin (BSA) in phosphate buffered saline (PBS) and a 64 well incubation gasket (FlexWell Grid, Grace Bio Labs) was attached. The slides were incubated with pooled mice serum diluted 1:100 or monoclonal antibodies (10, 100, 1000, 10000 ng/mL) in 1% BSA-PBS for 1 h at 37°C. After three washes with PBS containing 0.1% (v/v) Tween-20 (PBS-T) the slides were incubated with Alexa Fluor® 488 AffiniPure™ Rabbit Anti-Mouse IgG, Fcγ fragment specific (Jackson Immuno Research, Cat. 315-545-008), Alexa FluorTM 647 goat anti-mouse IgM (µ chain) (Invitrogen, Cat. A21238) diluted 1:400 for 1 h at 37°C. The slides were washed twice with PBS-T. After removing the gasket, the slides were washed once

with PBS and once with water. The dried slides were scanned with an InnoScan 1100 Fluorescence Scanner (Innopsys). Intensities from six individual spots per glycan were evaluated with Mapix Version 9.1.0 (Innopsys).

Fungal ELISA

An overnight culture of *Candida* was diluted to OD_{600}=0.1 in fresh YPD broth and grown for 4 h. Then, the cells were diluted to OD_{600}=0.6 in 10 mL PBS and washed once with PBS (8000 rpm, 5 min). Fixation with 500 µL 4% paraformaldehyde (Carl Roth, Cat. No P087.3) was performed overnight at room temperature. Cells were washed twice with PBS and resuspended in 10 mL PBS. High-binding 96-well polystyrene microtiter plates (Corning, Cat. No 3361) were coated overnight at 4°C with 50 µL of *Candida*. Each *Candida* strain was coated in separate wells of the ELISA plate. After washing the plates three times with PBS-Tween, they were blocked with 50 µL 1% BSA-PBS for 1 h at RT. The plates were either incubated with 50 µL pooled mice serum 1:100 dilution in 1% BSA-PBS or monoclonal antibodies (0.1, 10, 100 µg/mL) in triplicates at 37°C for 1 h. Control wells without sera or monoclonal antibodies were included. After three times washing with PBS-Tween, an incubation with 50 µL Goat IgG anti-Mouse IgG (Fc)-HRPO (Dianova, Cat. No 115-035-164) diluted 1:10000 in 1% BSA-PBS followed at 37°C for 1 h. Plates were washed thoroughly with PBS-Tween and developed using 100 µL HRP substrate 3,3',5,5'-tetramethylbenzidine (TMB) (Thermo Scientific, Cat. No 34028). Reactions were stopped with 100 µL 0.5M H_2SO_4 and the absorbance from three independent experiments was recorded at 450 nm using an ELISA plate reader instrument (CLARIOstar Plus, BMG Labtech). To account for background signal, all OD450 readings were normalized to the mean signal of secondary antibody-only control wells (no serum) of the corresponding *Candida* strain, which was subtracted from each measurement.

Correlation Analysis

To investigate the relationship between antibody levels, cytokine expression and fungal burden, **M13**-specific IgG levels (measured by glycan microarray) and cytokine concentrations (IFN-γ, IL-17A, IL-2) were compared with kidney and spleen fungal CFU counts. Pearson correlation analysis was performed using Microsoft Excel. Antibody and cytokine values were plotted against organ-specific fungal burdens and correlation coefficients (R) were calculated to assess the strength of association.

Hybridoma generation and antibody purification

Spleens were harvested from four immunized mice and splenocytes were fused with X63-Ag8.653 myeloma cells using PEG1500. Hybridoma generation was successful only with splenocytes from one animal; no viable hybridoma clones were obtained from the other three. Hybridoma clones were selected in HAT medium and screened by glycan microarray for specific binding to the synthetic glycan M13. A total of 38 wells tested positive. Clones were further selected based on two criteria: specific binding to M13 and IgG isotype. After three rounds of subcloning to ensure monoclonality, four hybridoma lines were expanded based on growth and antibody production. The three best-performing clones (7HF, 7KH, and 7CG), originating from different wells and plates but derived from the same animal, were purified from supernatant using protein A/G columns and quantified by NanoDrop.

Antibody characterization

Isotype analysis was carried out with a mouse monoclonal antibody isotyping kit test (#MMT1, Bio-Rad) following manufacturer's instructions. The purified mAbs were analyzed via SDS-PAGE gel analysis using 4-20% Tris-Glycine SDS-PAGE gel (Bio-Rad, Cat. No 4561094) under reducing and non-reducing conditions to confirm their purity. Confirmation of binding to original target antigen **M13** and epitope mapping was carried out via glycan microarray and binding to *Candida* was confirmed via ELISA and FACS. The variable regions of the monoclonal antibodies were sequenced following an established protocol [5]. The DNA sequences were translated into protein sequences and aligned using SnapGene (version 5.3.2) to

assemble the forward and reverse reads into full-length heavy or light chain variable regions. For immunogenetic annotation, sequences were submitted to IMGT/V-QUEST [6] (http://www.imgt.org/IMGT_vquest/) using the Mus musculus germline reference set. The tool was used to assign V gene segments and identify CDR1, CDR2 and CDR3 according to the IMGT numbering system. For multiple sequence alignment and comparison of the variable regions of the antibodies, the protein sequences were aligned with Clustal Omega via the EMBL-EBI web server (https://www.ebi.ac.uk/Tools/msa/clustalo/) using standard parameters. The aligned and annotated sequences are shown in Supplementary Figure 5 and Supplementary Table 1.

Fluorescent labeling of monoclonal antibodies

mAb 7CG was labeled using the DyLight 405 antibody labeling kit (Thermo Scientific, Cat. No 53020), mAb 7KH using DyLight 488 Antibody Labeling Kit (Thermo Scientific, Cat. No 53024) and mAb 7Hf using DyLight 550 Antibody Labeling Kit (Thermo Scientific, Cat. No 84530) according to the manufacturer's instructions. The dye-to-protein ratio was determined using a NanoDrop ND-1000 spectrophotometer (Thermo Fisher Scientific) and the labeled mAbs were aliquot and stored at -20°C. The mAb-dye conjugates were used in FACS analysis.

FACS

An overnight culture of *Candida* was diluted 1:10 and was grown for additional 4 h. 10 mL cells (OD_{600}=0.6) were washed twice with PBS and then fixed in 500 µL 4% paraformaldehyde o/n at 4°C. After washing the cells two times with PBS and resuspending them in 10 mL PBS, 100 µL of the culture were centrifuged and blocked with 1% BSA-PBS for 1 h at RT. Cells were washed once with PBS and incubated with anti-*Candida* mAb-dye conjugate at 100 µg/mL in 1% BSA-PBS. A control without mAb was prepared. Stained cells were then washed twice in PBS before final resuspension in Focusing Fluid (Thermo Fisher Scientific, Cat. No 4449791). Samples were analyzed using an Attune NxT flow cytometer (Thermo Fisher Scientific), with 100,000 events recorded for each sample from three independent experiments. The median fluorescence intensity was calculated for each sample using FloJo v.10.8.1 software. The gating strategy used is shown in Supplementary Figure 7.

Prophylactic infection model with glycoconjugate of systemic candidiasis

Eight to nine week-old immunocompetent female BALB/c mice (Charles River) were randomly assigned to groups (n = 6) and housed in IVC, with food and water provided ad libitum. Mice were immunized sub cutaneously (s.c.) with 100 µL of 1µg glycoconjugate or CRM_{197} in a mixture with 1:1 alhydrogel 2% adjuvant per mouse on days 1, 14 and 28. Mice were then intravenously (i.v.) via the tail vein route with $4x10^7$ cells/100 µL *C. auris* (NCPF13001#16) or sterile PBS per mouse at day 42. Mice were monitored and weighed at least once per day from day 1 up to day 45 and blood samples were taken on day 1, 28, 42 and 45. At day 45, animals were sacrificed via cardiac puncture and the kidneys and spleens were harvested for quantitative analysis of fungal burdens by plating on YPD agar (0.2 mg/mL gentamicin) (CFU/g tissue).

Prophylactic infection model with monoclonal antibodies of systemic candidiasis

Eight-week-old immunocompetent female BALB/c mice (Charles River) were randomly assigned to groups (n = 5) and housed in IVC, with food and water provided ad libitum. At 4 h prior to challenge, each mouse received 0.3 mg in 200 µL PBS of antibody 7HF or 7KH or sterile PBS by sub cutaneous (s.c.) injection. Each mouse was injected via the intravenous route (i.v.) with $1x10^7$ *C. auris* cells (NCPF13001#16) in 100 mL PBS or with sterile PBS. Then 24 h after infection, the mice received an additional 0.3 mg of antibody 7HF or 7KH in 200 µL PBS or sterile PBS by s.c. injection. The applied dose is within the range of previously reported *in vivo* studies using monoclonal antibodies targeting *Candida* species [7, 8]. Mice were monitored and weighed at least once per day from day 0 up to day 5 and serum was collected via tail

nick at 0, 28 and 100 h. After 100 h, animals were sacrificed via cardiac puncture and kidneys and spleens harvested for quantitation of fungal burdens on YPD plates (0.2 mg/mL gentamicin) after 36 h (CFU/g tissue).

Determination of cytokines

The sera were pooled by group and diluted 1:3 and was assayed in triplicates according to the manufacturer's instructions for commercial ELISA kits for cytokine content of IFN-γ (Invitrogen, Cat. No 88-7314), IL-2 (Invitrogen, Cat. No 88-7024) and IL-17A (Invitrogen, Cat. No 88-7371). Due to ethical restrictions regarding volume constraints in blood sampling from mice, cytokine levels were only measured at the end of the study and not before infection.

Lateral Flow Assay

The Universal Lateral Flow Assay kit (Abcam, Cat. No ab270537) was used to design and perform the Lateral Flow Assay (LFA), following the manufacturer's instructions. Briefly, the capture monoclonal antibody K was conjugated with the Ulfa-tag and the detection monoclonal antibody 7KH with 40 nm gold particles. Afterwards, the mAb 7KH-Ulfa was diluted in 1x Universal Running buffer with 0.1% bovine serum albumin to a final concentration of 25 µg/mL. The mAb 7KH-Gold was diluted in 1x Universal Running buffer with 0.1% bovine serum albumin to a final concentration of 6 OD. In addition, the 40 nm gold-biotin standard was also diluted in 1x Universal Running buffer with 0.1% bovine serum albumin to a final concentration of 1 OD. *Candida* cells were mixed with 1x Universal Running buffer with 0.1% bovine serum albumin to a final absorbance of OD_{600}=1. After 5 min incubation at room temperature of 5 µL diluted mAb 7KH-Ulfa, 5 µL diluted mAb K7KH-Gold, 5µL diluted Gold-biotin and 75 µL *Candida* suspension, 80 µL were loaded on the sample pad. The presence of Control line and Test line was determined visually after 20 min and recorded with ImageQuant™ LAS 4000 mini (GE Healthcare Bio-Sciences AB).

Statistical Analysis

Results were analyzed using GraphPad Prism 10.4.0 (GraphPad Software, Inc.). All graphs depict mean ± SEM. Differences were tested for significance using the statistical tests stated in the respective figure legend.

Supplementary Figures

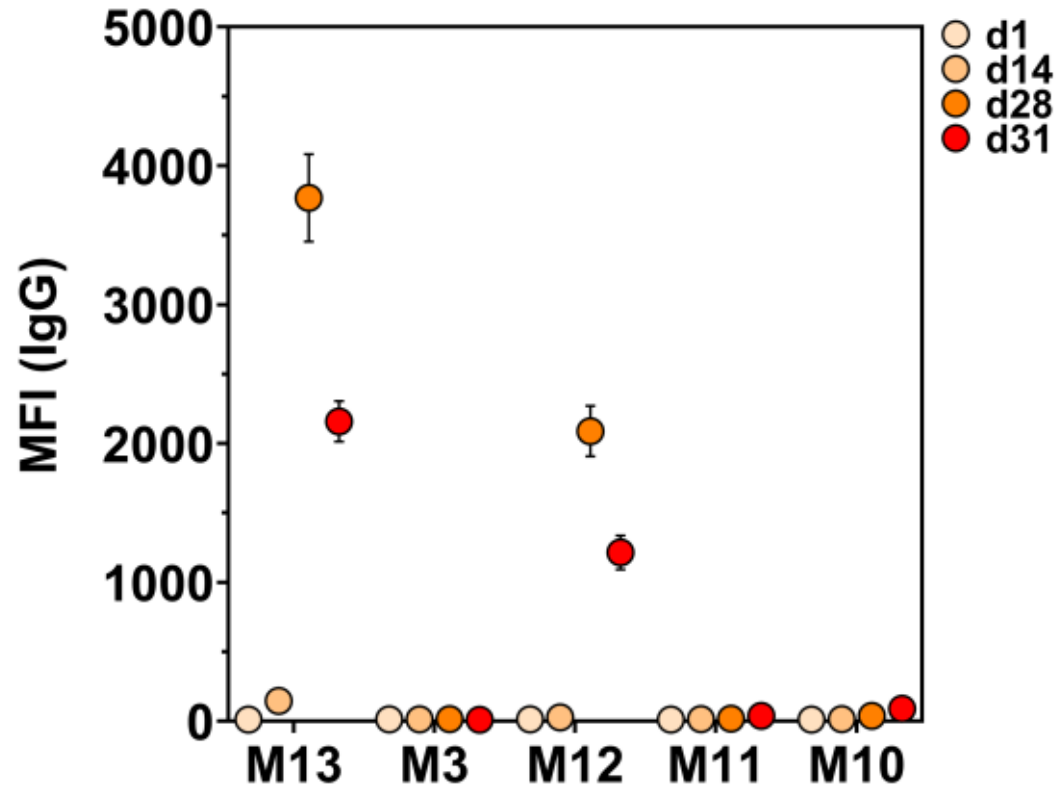

Figure S1. Epitope specificity of IgG antibodies in sera collected pre- (day 1) and post-immunization (days 14, 28, and 31) with the **M13**-CRM197 glycoconjugate. Binding to synthetic glycans **M13**, **M12**, **M11**, **M10**, and **M3** was assessed by glycan microarray analysis. Specific recognition of **M13** and related glycans emerged starting at day 28, indicating the development of a glycan-specific IgG response following immunization.

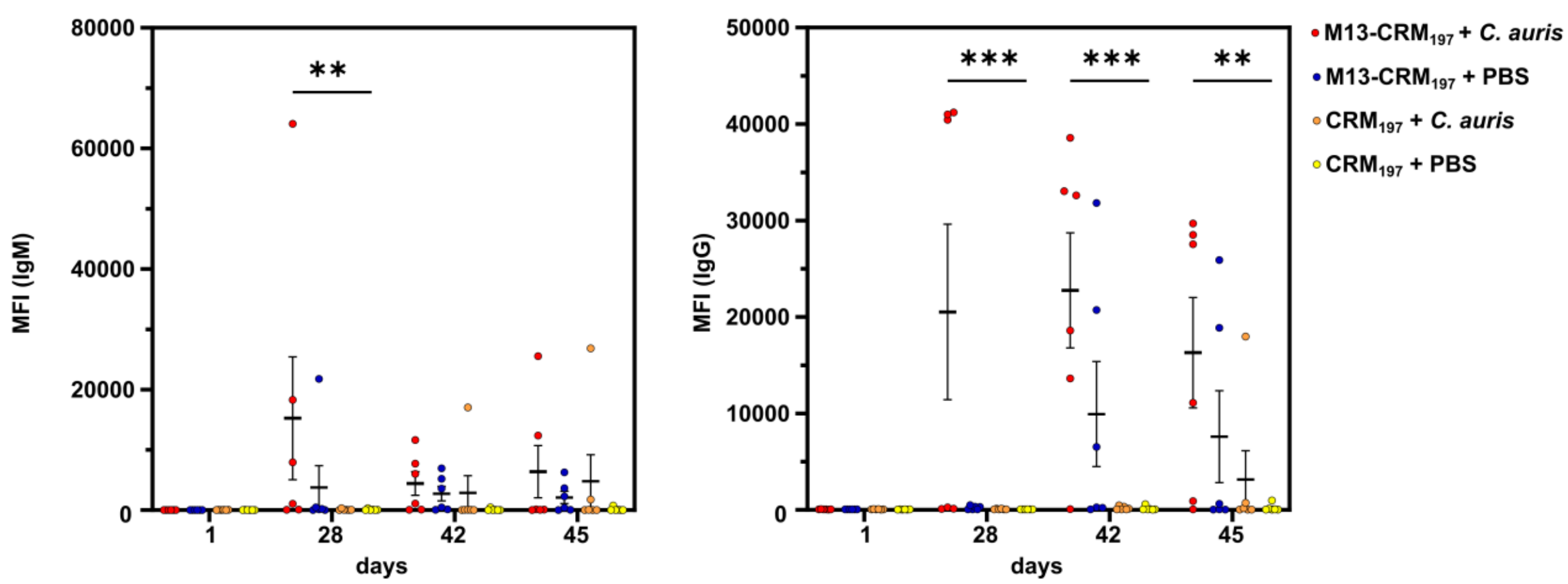

Figure S2. Glycan-specific antibody responses in the *in vivo* protective efficacy of the glycoconjugate **M13**-CRM197 against *C. auris* infection study. Glycan microarray analysis showing mean fluorescence intensity (MFI) of serum IgM and IgG antibodies specific to **M13** on day 1 (pre-immunization), 28, 42 (day of infection) and 45 (post-immunization). Data represent antibody response of single animals (*n=6* per group) and mean ± SEM. Statistical significance was determined using two-way ANOVA test with (***) p <0.0002 and (**) p<0.0021.

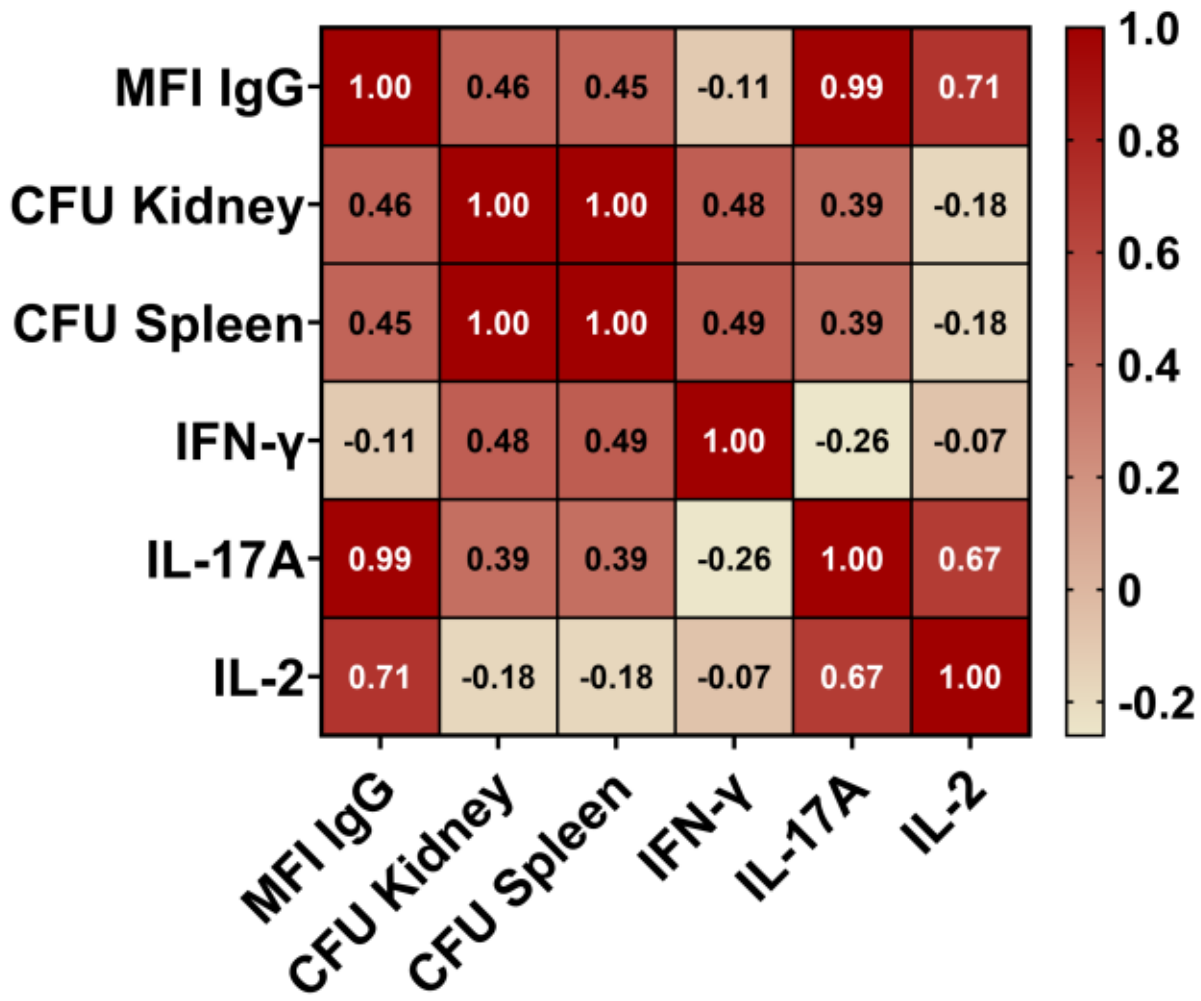

Figure S3. Correlation matrix of antibody levels, fungal burden and cytokine responses. Pearson correlation coefficients were calculated between **M13**-specific antibody levels, CFU counts in kidney and spleen and serum cytokine concentrations (IFN-γ, IL-17A, IL-2) at study endpoint (day 45) in the *in vivo* protective efficacy of the glycoconjugate **M13**-CRM197 against *C. auris* infection study. Data are visualized as a heatmap using conditional formatting. Strong positive and negative correlations are indicated by color gradients.

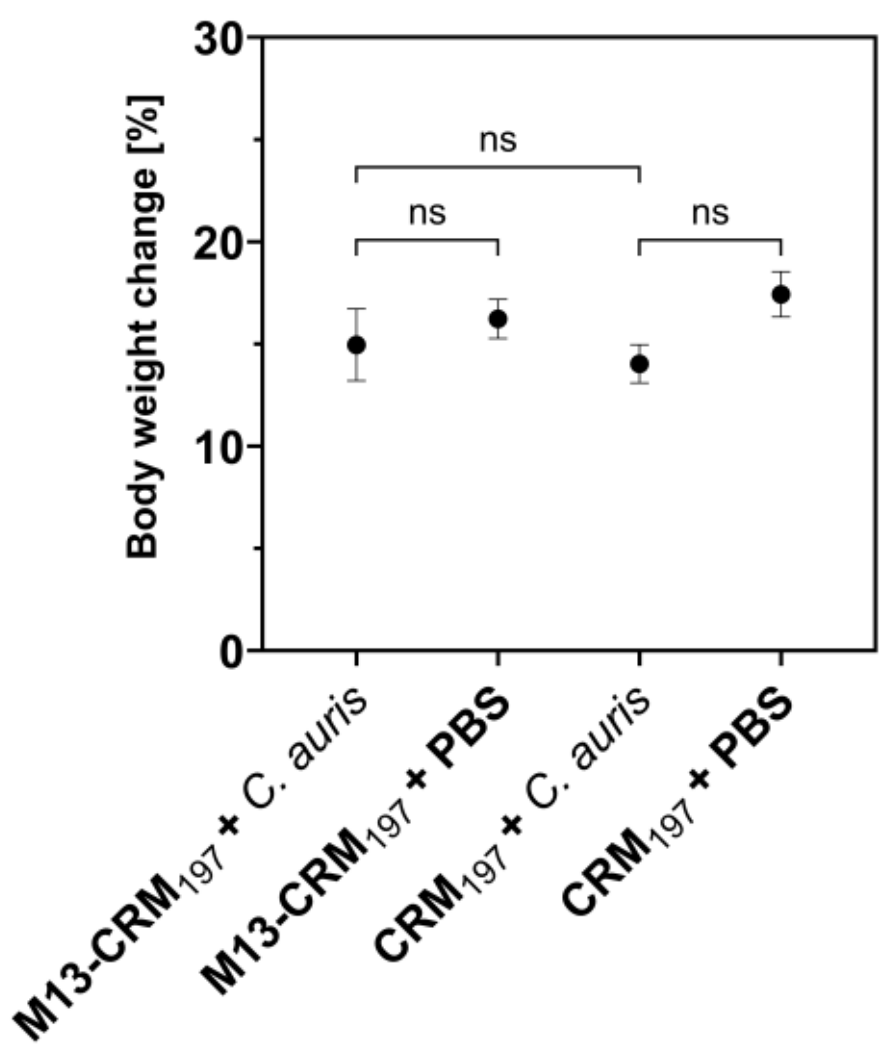

Figure S4. Body weight change following active immunization with the **M13**-CRM197 glycoconjugate. Body weights were recorded on day 1 (pre-infection) and day 45 (endpoint). Percent weight change per mouse is shown (*n=6*). Data represent mean ± SEM. Statistical analysis was performed using one-way ANOVA with Tukey's multiple comparisons test. ns = not significant.

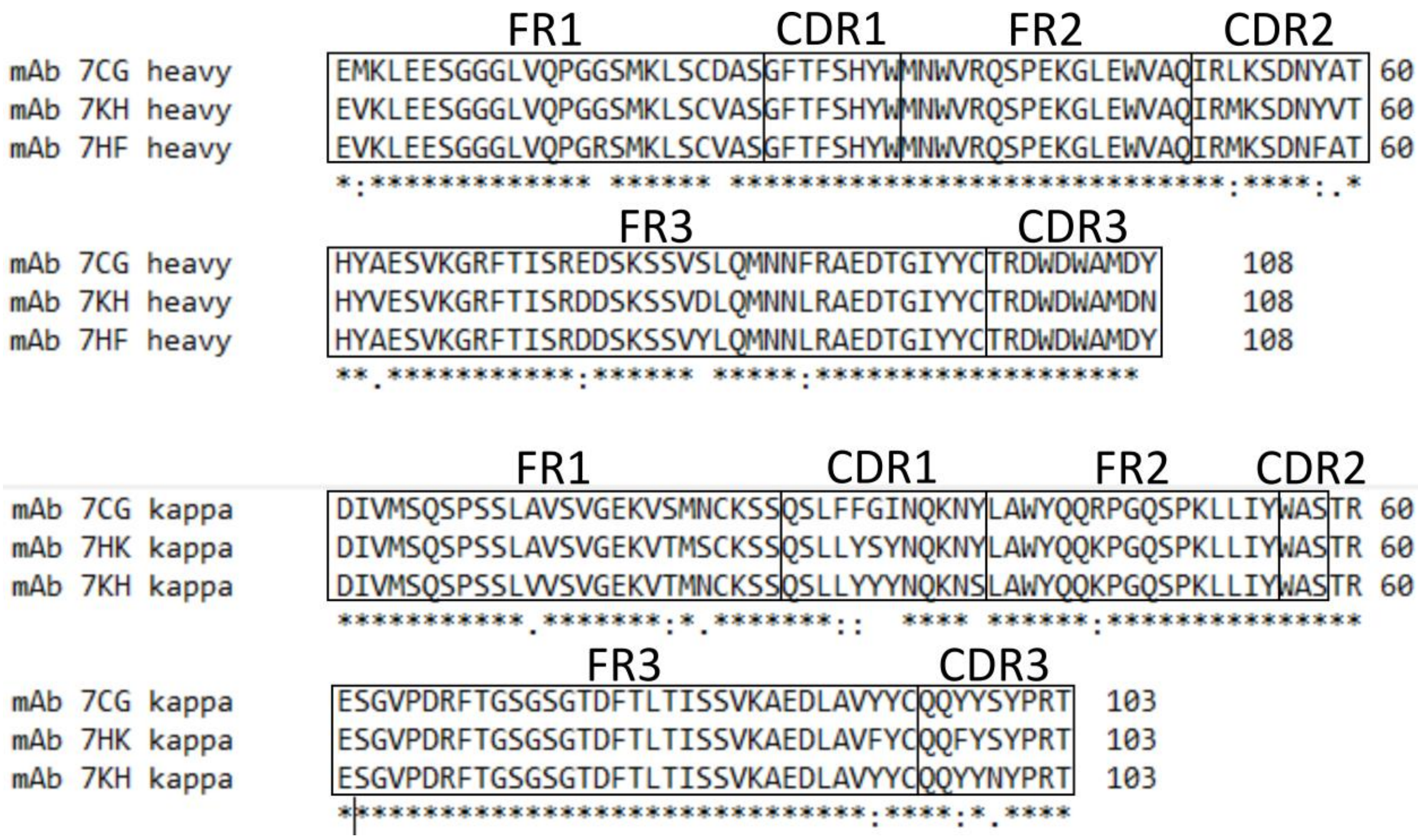

Figure S5. Alignment of variable regions sequences of mAb 7CG, 7KH, 7HF heavy and kappa chain. FR= Frame region, CDR= Complementarity-determining region, *= identical amino acid.

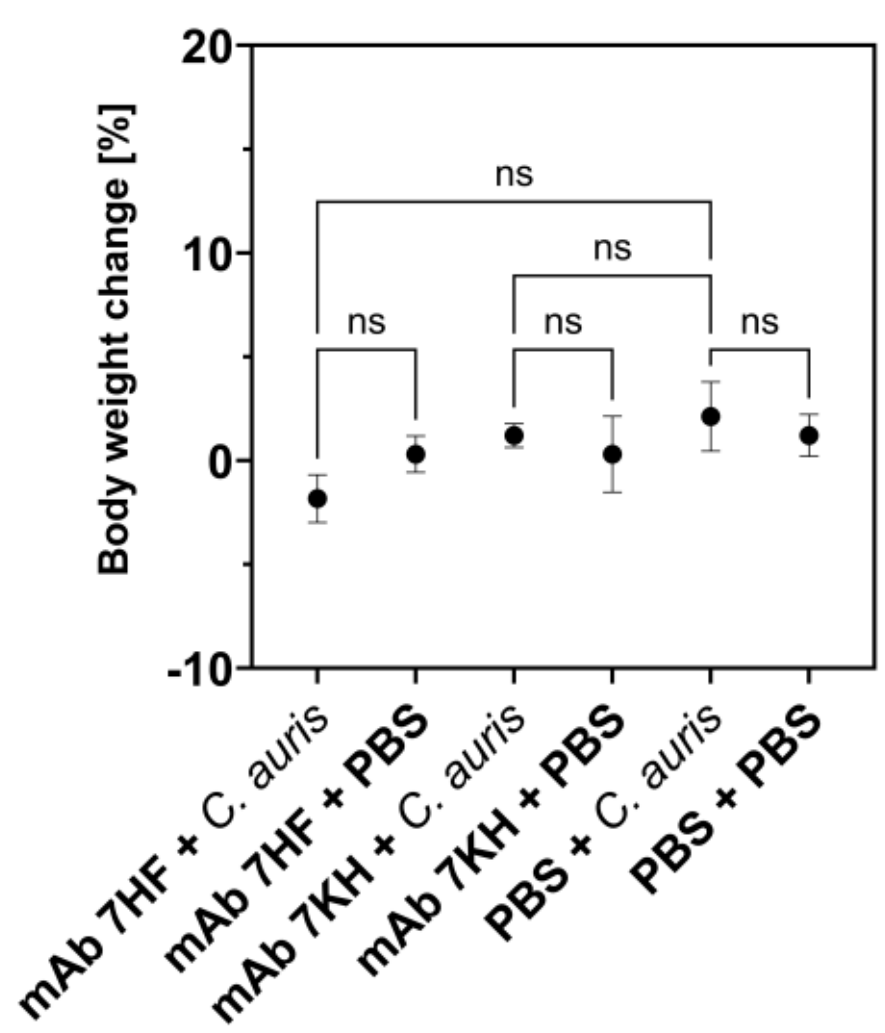

Figure S6. Body weight change following passive immunization with monoclonal antibodies. Body weights were recorded on day 1 (pre-infection) and day 5 (endpoint). Percent weight change per mouse is shown (*n=5*). Data represent mean ± SEM. Statistical analysis was performed using one-way ANOVA with Tukey's multiple comparisons test. ns = not significant.

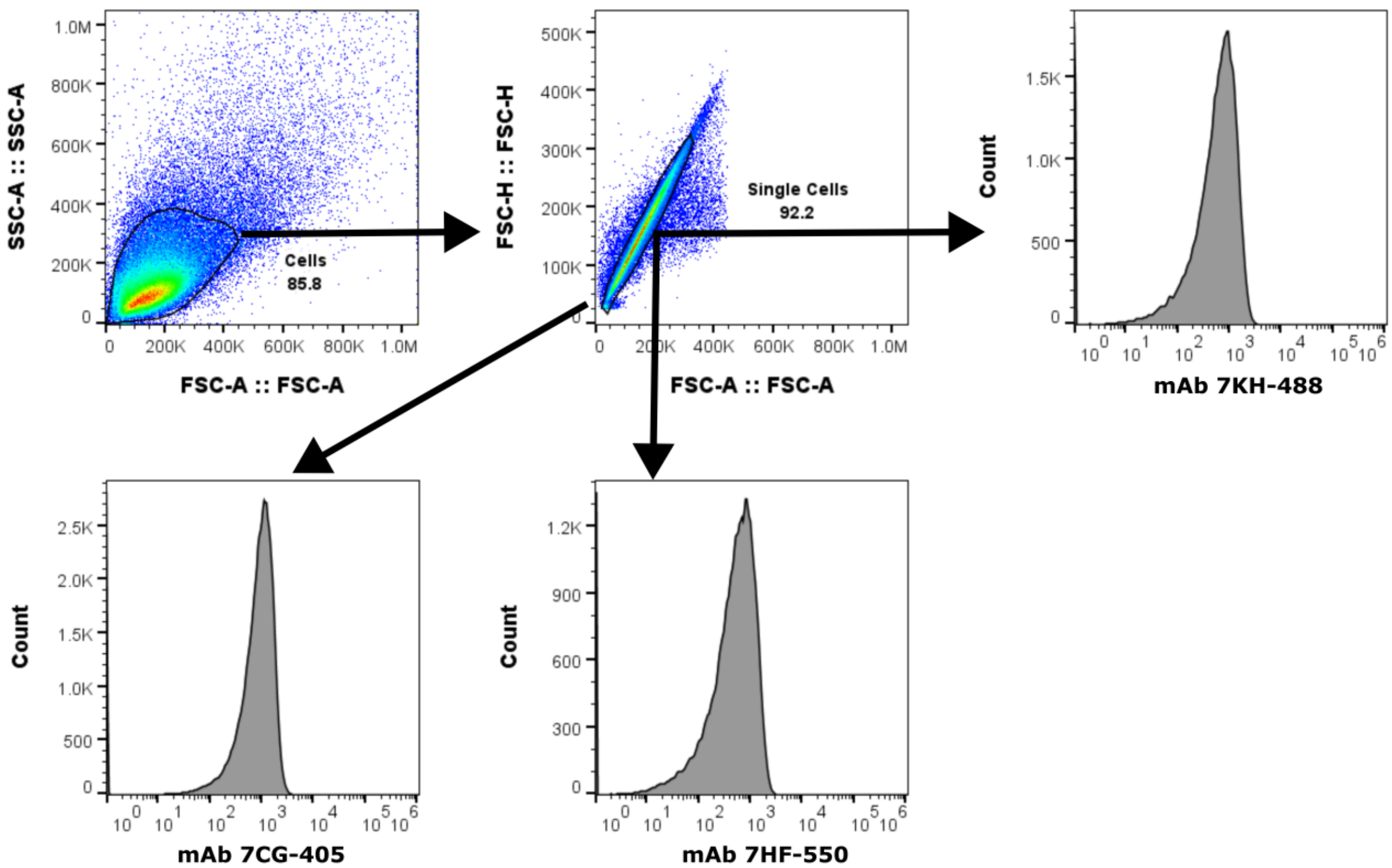

Figure S7. Gating strategy used to determine binding of mAbs to *Candida* cells via FACS. The median fluorescence intensity was determined from the single cell population for mAb 7KH-488, 7CG-405 and 7HF-550. An example of unstained cells is shown.

Table S1. Immunogenetic annotation of monoclonal antibodies 7CG, 7KH and 7HF. Heavy and light chain variable regions were analyzed, identifying germline V, D, and J gene segments.

Antibody	Heavy V gene	Heavy D gene	Heavy J gene	Light V gene	Light J gene
**7CG**	IGHV6-3*01	IGHD4-1*01	IGHJ4*01	IGKV8-30*01	IGKJ1*01
**7KH**	IGHV6-3*01	IGHD4-1*01	IGHJ4*01	IGKV8-30*01	IGKJ1*01
**7HF**	IGHV6-3*01	IGHD4-1*01	IGHJ4*01	IGKV8-30*01	IGKJ1*01

Dataset S1: Glycan microarray raw data of immunization study with glycoconjugate M13-CRM_{197}. Raw fluorescence intensity values from glycan microarray analysis showing IgG and IgM antibody responses toward the synthetic glycan M13 and related structures at various time points following immunization with the glycoconjugate M13-CRM_{197}. Data are presented for individual mice and include measurements from pre-immunization (Day 1) and post-immunization (Days 14, 28 and 31).

Dataset S2: ELISA raw data of binding of IgG antibodies in sera of glycoconjugate immunization study to different *Candida*. Raw absorbance values (450 nm) from ELISA experiments showing binding of pooled mouse sera (from 4 immunized mice) to fixed *Candida* cells representing different clades. Data were normalized to the signal obtained with secondary antibody control wells (no primary antibody).

Dataset S3: Glycan microarray analysis in *in vivo* challenge following glycoconjugate immunization. Raw fluorescence intensity data from glycan microarray analysis of serum IgG and IgM antibodies collected at

multiple time points (day 1, 28, 42 and 45) during the immunization and infection study with the M13-CRM_{197} glycoconjugate.

Dataset S4: Fungal burden (CFU) in *in vivo* challenge following glycoconjugate immunization. Raw data of CFU per organ (kidney and spleen) from individual mice in the *in vivo* challenge study with *C. auris* following immunization with M13-CRM_{197} glycoconjugate. Each row represents a single animal. Data are presented as absolute CFU counts recovered from organ homogenates after plating and incubation.

Dataset S5: Cytokine measurements in the *in vivo* challenge following glycoconjugate immunization. Raw cytokine data (IFN-γ, IL-17A, IL-2) from pooled serum samples collected at the endpoint of the *C. auris in vivo* challenge following immunization with M13-CRM_{197}. Each cytokine was measured using ELISA. Shown are the absorbance values (450 nm), the standard curves used for quantification and the resulting calculated cytokine concentrations (in pg/mL) based on the standard curve.

Dataset S6: Body weight in *in vivo* challenge following immunization with M13-CRM_{197} glycoconjugate. Raw body weight measurements of mice at the start (Day 0) and end (Day 45) of the *in vivo* challenge study following immunization with M13-CRM_{197} glycoconjugate. Data include group allocation, mouse ID, body weight at both time points, weight change in grams, and percentage change.

Dataset S7: Glycan microarray raw data for monoclonal antibodies. Raw glycan microarray data showing IgG binding of monoclonal antibodies (7HF, 7KH and 7CG) to synthetic glycans. Data include fluorescence intensity values for each antibody across the glycan panel in different concentrations.

Dataset S8: ELISA binding data of monoclonal antibodies. Raw ELISA absorbance data showing IgG binding of monoclonal antibodies (7HF, 7KH, and 7CG) to *Candida* at different concentrations. Absorbance values were normalized against secondary antibody-only controls to correct for background.

Dataset S9: Flow cytometry (FACS) binding data of monoclonal antibodies. Raw flow cytometry data showing binding of monoclonal antibodies 7HF, 7KH and 7CG to fixed *Candida* cells. Data include median fluorescence intensity (MFI) values from three independent experiments. Background staining was controlled using secondary antibody only.

Dataset S10: Fungal burden (CFU) in *in vivo* challenge following monoclonal antibody treatment. Raw CFU counts from kidney and spleen homogenates of mice treated with monoclonal antibodies 7HF, 7KH, or control (PBS) in the passive immunization model. Each row represents individual animals, with fungal burden recorded as CFU per organ.

Dataset S11: Cytokine levels in *in vivo* challenge following monoclonal antibody treatment. Raw data of cytokine measurements (IFN-γ, IL-17A and IL-2) from serum samples collected after passive immunization with monoclonal antibodies 7HF, 7KH, or PBS control. Values represent concentrations calculated from absorbance readings using a standard curve.

Dataset S12: Body weight in in vivo challenge following monoclonal antibody treatment. Raw body weight measurements of mice at the start (Day 0) and end (Day 5) of the *in vivo* challenge study following treatment with monoclonal antibodies. Data include group allocation, mouse ID, body weight at both time points, weight change in grams, and percentage change.

[1] R. K. Singh, E. E. Reuber, M. Bruno, M. G. Netea, P. H. Seeberger, *Chem Sci* **2023**, *14*, 7559-7563.

[2] R. K. Singh, *Chemical Synthesis of Oligosaccharides as Basis for the Development of Carbohydrate-Based Vaccine Candidates against Streptococcus suis Serotype 18 and Candida auris*, Freie Universitaet Berlin (Germany), **2023**.

[3] M. P. Lisboa, N. Khan, C. Martin, F. F. Xu, K. Reppe, A. Geissner, S. Govindan, M. Witzenrath, C. L. Pereira, P. H. Seeberger, *Proc Natl Acad Sci U S A* **2017**, *114*, 11063-11068.

[4] P. N. Papapanou, M. Sanz, N. Buduneli, T. Dietrich, M. Feres, D. H. Fine, T. F. Flemmig, R. Garcia, W. V. Giannobile, F. Graziani, H. Greenwell, D. Herrera, R. T. Kao, M. Kebschull, D. F. Kinane, K. L. Kirkwood, T. Kocher, K. S. Kornman, P. S. Kumar, B. G. Loos, E. Machtei, H. Meng, A. Mombelli, I. Needleman, S. Offenbacher, G. J. Seymour, R. Teles, M. S. Tonetti, *J Periodontol* **2018**, *89 Suppl 1*, S173-S182.

[5] L. Meyer, T. Lopez, R. Espinosa, C. F. Arias, C. Vollmers, R. M. DuBois, *PLoS One* **2019**, *14*, e0218717.

[6] V. Giudicelli, X. Brochet, M. P. Lefranc, *Cold Spring Harb Protoc* **2011**, *2011*, 695-715.

[7] S. Palliyil, M. Mawer, S. A. Alawfi, L. Fogg, T. H. Tan, G. B. De Cesare, L. A. Walker, D. M. MacCallum, A. J. Porter, C. A. Munro, *Antimicrob Agents Chemother* **2022**, *66*, e0195721.

[8] E. G. Youssef, L. Zhang, S. Alkhazraji, T. Gebremariam, S. Singh, N. Y. Yount, M. R. Yeaman, P. Uppuluri, A. S. Ibrahim, *Front Immunol* **2020**, *11*, 76.
